# Multiplex DNA fluorescence in situ hybridization to analyze maternal vs. paternal *C. elegans* chromosomes

**DOI:** 10.1186/s13059-024-03199-6

**Published:** 2024-03-14

**Authors:** Silvia Gutnik, Jia Emil You, Ahilya N. Sawh, Aude Andriollo, Susan E. Mango

**Affiliations:** 1 Biozentrum, University of Basel, 4056 Basel, Switzerland; 2 Current address: University Children’s Hospital Zürich, Pediatric Oncology and Children’s Research Center, Balgrist Campus AG, Lengghalde 5, 8008 Zürich, Switzerland; 3 Current address: Department of Biochemistry, University of Toronto, Toronto, ON M5G 1M1 Canada

**Keywords:** Chromosome tracing, Embryo, DNA FISH, Chromosome pairing, Parent-of-origin

## Abstract

Recent advances in microscopy have enabled studying chromosome organization at the single-molecule level, yet little is known about inherited chromosome organization. Here we adapt single-molecule chromosome tracing to distinguish two *C. elegans* strains (N2 and HI) and find that while their organization is similar, the N2 chromosome influences the folding parameters of the HI chromosome, in particular the step size, across generations. Furthermore, homologous chromosomes overlap frequently, but alignment between homologous regions is rare, suggesting that transvection is unlikely. We present a powerful tool to investigate chromosome architecture and to track the parent of origin.

**Supplementary Information:**

The online version contains supplementary material available at 10.1186/s13059-024-03199-6.

## Background

When an egg and sperm fuse to generate a new organism, not only genetic information in the form of the paternal and maternal genome is passed to the next generation. Oocytes and sperm contribute non-genetic factors, ranging from modified histones and DNA methylation to RNA and proteins. These maternally and paternally contributed factors are crucial for the early stages of development and dictate translational or transcriptional regulatory steps [1–3]. In addition, parental factors influence nuclear organization and transcription [4–8]. For example, in *C. elegans*, modified histones H3K27me3, H3.3, H3K4me2, and H3K9me3 are transmitted from parents to offspring [9–12], while a host of chromatin factors influence the memory between generations by emerging mechanisms [7, 13, 14]. Other species also show sex-specific, parental effects on chromatin regulation. Striking examples include differential expression of the IGF2-H19 locus in mammals, silencing of the paternal X chromosome in marsupials and the early mouse embryo [15], and loss of the paternal genome in the males of many arthropod species [16]. DNA methylation, long non-coding RNAs, and the Polycomb complex have been implicated in these processes and can give rise to altered chromosome looping and genome organization [17].

Analysis of parent-of-origin effects typically relies on sequence differences between alleles. RNAs or genes bearing polymorphisms are used to distinguish the parental origins of the homologous chromosomes. One downside of this approach is that loci that lack polymorphisms cannot be studied, which restricts analysis to a smaller cohort of genes [18]. Another downside is that sequencing errors can generate inaccurate estimates of biased allele expression [19]. Here we describe an approach that circumvents these difficulties by separating parental identification that relies on polymorphisms, from analysis of chromosome organization, which does not.

*C. elegans* nuclei face a complex organizational problem, whereby 100 Mb of genomic DNA is packaged into a confined space as small as ~1 µm in diameter. A large body of work has discovered layers of chromatin organization that include DNA loops, topological associating domains (TADs), and compartments in multiple organisms [20]. TADs reflect contiguous sequences that show physical interactions due to loop extrusion and range in size from 20 kb to 1 Mb. They can regulate interactions between cis-regulatory regions and target promoters [21], but seemingly at only some regions of the genome [22]. Compartments are non-contiguous sequences that associate based on the transcriptional activity and histone modifications within those sequences [23, 24]. In *C. elegans*, multi-megabase-scale compartments form gradually, during gastrulation [25] and organize the chromosome into a B-A-B configuration [24, 26]. Small TADs are found along the chromosome, encompassing ~3 genes per TAD, and these domains interact to form small, polycomb-dependent compartments [27, 28]. These structures have been seen in one strain of *C. elegans*, called N2, but whether these structures exist across multiple strains is not known.

To define loops, TADs, and compartments, many studies rely on sequencing-based methods that average the signal from thousands or millions of nuclei. Haplotype-resolved HI-C methods enable parental chromosomes to be distinguished within a diploid cell, but usually require averaging many chromosomes together [29]. To circumvent this difficulty, we have focused on chromosome tracing, which tracks chromosome and sub-chromosome organization at the level of single molecules. This method relies on iterative DNA fluorescence in situ hybridization (FISH) for a molecular connect-the-dots approach [23, 30, 31]. Normally, chromosomes in diploid cells are distinguished by marking the two chromosome territories within the nucleus, but currently one cannot determine which chromosome is derived from sperm and which from oocytes. Here we extend our chromosome tracing method [25] to differentiate maternal and paternal chromosomes. Our strategy relies on F1 hybrid offspring from crosses of two closely related *C. elegans* strains, Bristol (N2) and Hawai’ian (HI), and utilizes their divergent genomic sequences to distinguish each chromosome territory. To analyze these hybrids accurately, we have designed and implemented strain-specific FISH probe sets and developed an image analysis pipeline that accurately distinguishes the parental chromosomes in embryos. A benefit of our method is that the probes are used to identify the chromosome territory associated with a particular genotype and not for tracing per se*.* Therefore, the tracing probes are not restricted to regions of the genome with strain-specific differences, nor do they rely on sensitive differences in hybridization temperature due to single-molecule polymorphisms. Using this new approach, we show a proof of concept by determining the chromosome conformation of wild-type maternal and paternal, N2 and HI for chromosome V. We define the degree of overlap between pairs of chromosomes and find that chromosomes intermingle frequently, but only rarely pair. In addition, we show that N2 and HI conformations are overall similar, despite genetic differences between the two strains.

## Results

### N2- and HI-specific probes selectively mark their respective chromosomes

**Fig. 1:**
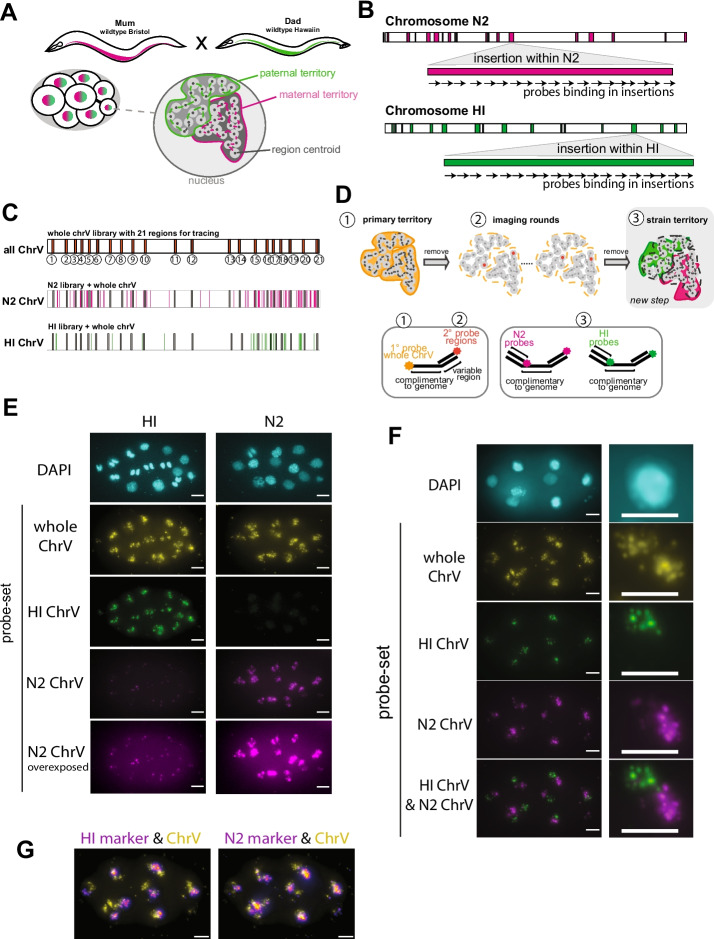
Haplotype-resolved chromosome tracing in *C. elegans*. **A** Schematic of crossing experiments. **B** Schematic of probe design strategy. **C** Location of whole-chromosome V (ChrV) tracing library with 21 regions and N2 and HI libraries interspersed along ChrV. **D** Schematic representation of the imaging workflow using an automated imaging system (see “Methods”), and probe design used for the indicated steps. **E** DNA Fish on embryos from N2 and HI, respectively, using the whole ChrV library, N2 ChrV, and HI ChrV-marking libraries. Note that the N2 ChrV libraries cause a small punctual background staining in HI. Scale bar, 5µm. **F** DNA FISH on embryos derived from crosses between N2 hermaphrodites and HI males, using the whole ChrV library, N2 ChrV, and HI ChrV-marking libraries. The haplotypes are well distinguishable. Scale bar, 5µm. **G** Overlay of ChrV territory signal with HI marker (left) and N2 marker (right) in the embryo used in **F**. Scale bar, 5µm

To distinguish maternally vs paternally derived chromosomes within a single nucleus, we focused on F1 hybrid offspring from crosses between divergent *C. elegans* strains (Fig. 1A). We chose N2, the commonly used laboratory strain, and the related HI as crossing partners for four reasons: (1) the two strains can interbreed; (2) they have been extensively characterized at the sequence level [32–35]; (3) HI is one of the most divergent *C. elegans* isolates from N2, with over 170,000 SNPs between the two strains as well as many insertions and deletions, which supplied regions across the two genomes to design strain-specific chromosome marking probes [32, 36–38]; (4) the two strains show a high level of synteny and are similar enough in sequence that a set of common chromosome tracing probes could be used to trace both N2 and HI chromosomes, keeping reagent costs low.

In conventional chromosome tracing of *C. elegans* embryos, fluorescently labeled primary DNA FISH probes are hybridized to defined regions along a chromosome, such as the 22 regions along ChrV [26]. Due to a fluorophore present on each whole-chromosome tracing probe, when imaged en masse, the probes reveal the chromosome territory (Fig. 1D, panel 1). Next, fluorescent region-specific probes are hybridized sequentially to readout tails on the primary probes, to visualize individual locations along ChrV (Fig. 1D, panel 2). This method allows one to determine the structure of individual chromosomes in 3D by a molecular connect-the-dots approach.

We modified the chromosome tracing method in two ways for our strain-specific approach. First, in addition to the common whole-chromosome tracing probes, we hybridized the strain-specific N2 and HI probes to the F1 hybrid embryos. In contrast to the common whole-chromosome tracing probes, we generated the strain-specific probes without a fluorophore, but with tails to bind two secondary oligos. The same binding sites for two secondary oligos were present on all probes for N2, and for a different secondary oligo binding site on all probes for HI. This design provided a means to label the strain territory by on-microscope hybridization using the N2-specific secondary oligos and the HI-specific secondary oligos equipped with distinct fluorophores, during image acquisition. We note that restricting fluorophores to the secondary probes gave flexibility regarding the choice of fluorophore for each experiment and lowered the cost of probe synthesis. Second, some regions along ChrV had few possible strain-marking probes due to a lack of strain-specific insertions, and therefore we decided to increase the signal by equipping each N2- and HI-specific probe with two binding sites for secondary oligos. We imaged the N2 and HI markers at the end of the chromosome tracing experiment (Fig. 1D, panel 3). We note, however, that since fluorescent labeling of the strain markers relies on on-stage hybridization during image acquisition, the strain markers could be imaged any time after primary probe imaging.

We made use of previously annotated insertions and deletions within the N2 and HI genomes [32] to design probes that were specific for one strain and that, together, could distinguish HI and N2 chromosome territories (Fig. 1B). We focused on insertions larger than 1000 nucleotides (nts), which would accommodate a minimum of 33 potential 30-mer probes and provided a strong signal to noise ratio for DNA FISH. Across both genomes, each chromosome harbored a varying number of inserted regions >1000 nts, from a high of 172 on ChrV to a low of 37 on the X chromosome (Additional File 1: Figure S1A), where N2 contained more insertions of >1000 nts across all chromosomes compared to HI. The total length of inserted sequences >1000 nts was the highest for ChrV (Additional File 1: Figure S1B), and we therefore decided to focus on ChrV to illustrate the proof of concept. We designed suitable strain-marking probes to N2 and HI, using a previously described probe design method [26, 39] (see “Methods”). This approach resulted in 5577 probes for N2 ChrV and 1831 for HI ChrV, where each probe was unique to one locus and predicted to bind exclusively to one genome. The strain-marking probes could hybridize along the length of the respective chromosome and were interspersed with the shared tracing probes for ChrV (Fig. 1C & Additional File 1: Figure S1C).

To test the specificity of the N2 and HI probes and assure compatibility of the new probe sets with the shared tracing probe set, we hybridized all three probe sets to fixed homozygous N2 and HI (CB4856) embryos. As expected, the shared tracing probe set for ChrV enabled visualization of ChrV both in N2 and HI homozygous animals (Fig. 1E). HI-specific probes marked the HI chromosome without detecting a signal from N2. The N2 probes detected the N2 chromosome robustly. In addition, we observed a faint signal from the HI strain using the N2 strain probes (Fig. 1E). This signal likely corresponds to a small region within the HI genome that was not included in the Thompson HI genome, which we used for our probe design, but which was present in a later HI genome release, as revealed by a BLAST search [32, 38] (see “Methods”). Nevertheless, we found that this low signal did not interfere with image segmentation and chromosome classification, in subsequent experiments (see below). Future libraries could remove these sequences.

To assess if the strain-specific probe sets perform well with heterozygous embryos, we mated N2 mothers with HI fathers. The two chromosomes were clearly visible, and N2 and HI probes marked their respective chromosome, allowing us to determine the parent of origin for each chromosome (Fig. 1F). Similarly, when mating HI mothers with N2 fathers, haplotypes were clearly distinguishable (Additional File 1: Figure S1D). As expected, the N2 and HI signals overlapped partially with the shared ChrV territory signal (Fig. 1G), which reflects the overlap between the shared and strain-specific probe sets along ChrV. Together, it is clear which chromosome comes from which strain (Fig. 1F). We conclude that the N2 and HI probe sets can distinguish ChrV derived from N2 or HI.

### N2 and HI chromosomes form barbells, but HI is more compact

N2 and HI are both *C. elegans*, but they harbor sequence differences, some of which are predicted to affect chromatin architecture [32, 38, 40]. For example, HI lacks the germline RNAi component *ppw-1* [41] and the sperm-expressed, selfish genetic element *peel-1* [42]*.* N2 and HI also differ in certain multi-gene families such as BATH factors (BTB/POZ + MATH domains) and nuclear hormone receptors [32]. The effects of all but *ppw-1* are unknown. Since experimentally introduced siRNAs are sufficient to induce chromatin compaction, it was possible that the lack of germline RNAi in HI strains might influence chromosome conformation [41, 43]. We therefore wanted to investigate if these two strains exhibited similar genome structures by comparing chromosomes from N2 or HI homozygous embryos.

One method to assess a chromosome’s compaction and 3D path in nuclear space is to compare the genomic (2D) and spatial (3D) pairwise distance measurements using power-law fitting [23, 26, 44]. Power-law fitting takes into account the polymer nature of the chromosome (where regions close in genomic distance are expected to be close in spatial distance, and vice versa). We can derive metrics from the fit of the raw data to describe the polymer step size (compaction) and scaling exponent (3D path). The step size is the average distance between the points along the chromosome. A smaller step size indicates a more compact chromosome. The scaling exponent of a random-walk polymer is expected to be 0.5, while an ideal highly crumpled (fractal globule) polymer where all spatial distances are proportional to genomic distances has a scaling exponent of 0.3. Our prior studies found that *C. elegans* chromosomes had a scaling exponent of ~0.2, which reflects more intermixing between points with large genomic distances [26].

**Fig. 2:**
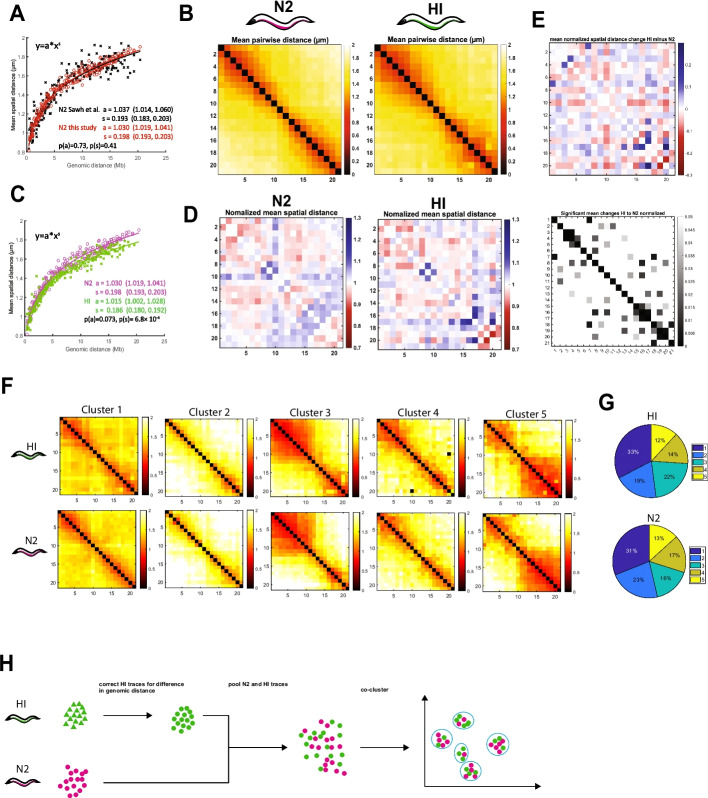
Highly similar N2 and HI ChrV organization. **A** Power-law fits of mean pairwise distances in µm versus genomic distance in Megabases (Mb) for N2 data generated in this study (black) and previously (red) [26]. s = scaling exponent of spatial distance, a = step size. Values in brackets are 95% confidence intervals of the fit. *P*-values are acquired from a linear regression analysis of log-transformed data. **B** ChrV mean distance matrix for N2 (left) and HI (right) in early embryos (2–40 cells), colored by distance (in μm). *N* = 6072 (N2) & *N* = 5905 (HI). **C** Power-law fits of mean pairwise distance for N2 data (magenta) and HI data (green). s = scaling exponent of spatial distance, a = step size. Values in brackets are 95% confidence intervals of the fit. *P*-values are acquired from a linear regression analysis of log-transformed data. **D** Normalized spatial distances for N2 date (left) and HI data (right). Data is plotted as observed over expected. The expected spatial distance is determined by the fit to the power-law function shown in **C**. Red marks regions that are closer together than expected from the fit and blue regions that are further away. The right panel shows the significant mean changes. **E** Changes between normalized spatial distances of N2 and HI data (left) and *p*-value of the spatial distance changes. Red marks regions that are closer together than expected from the fit and blue regions that are further away. **F** Mean pairwise distances in µm of subpopulations of chromosome conformations for N2 and HI traces as determined by unsupervised clustering. N2 and HI traces were pooled as outlined in **H**. **G** Distribution of traces within clusters from **F** shows both strains have similar cluster frequencies. Chi-square statistic was 29.94, *p*-value = 5.03 × 10^−6^ and Cramér’s *V* = 0.08. **H** Strategy for co-clustering to determine statistical similarity. Axis numbering in **B–F** represents positions across the chromosome as shown in Figure 1C

We examined homozygous N2 or HI embryos that were at or below the 40-cell stage (Additional File 1: Figure S2A). We found that the newly derived N2 conformation was virtually identical to the previously published average N2 configuration for ChrV [26], with a comparable step size (1.030 vs. 1.037) and scaling exponent (0.198 vs. 0.193) when fitted to a power-law function (Fig. 2A). The nascent B compartments (or “chromosome arms” at TADs 1-7 and 18-22) exhibited long-range folding, whereas the nascent A compartment, encompassing TADs 8-17, was more extended (Fig. 2B). This result revealed the reproducibility of chromosome tracing in *C. elegans*, with little batch-to-batch variation between independent studies.

The HI chromosome showed similar overall properties to N2, with compacted chromosome arms and an extended center (Fig. 2B). HI ChrV was slightly more compact than N2, as revealed by its smaller step size in power-law fitting (1.015 for HI vs. 1.030 for N2) (Fig. 2C); however, these differences were not statistically significant. N2 and HI also had differences in their scaling coefficients (0.198 for N2 and 0.187 for HI), which means that increasing genomic distances produced lower growth of spatial distances in HI compared to N2. These differences between N2 and HI may reflect the smaller size of the HI genome, which is ~2 Mb shorter than the N2 genome or approximately 2% of the total [32]. In addition, the relative size difference between chromosomes from HI and N2 is largest for ChrV, with HI ChrV being 741kb shorter than the N2 ChrV, representing a difference of 3.5% [32]. The differences in scaling coefficient and step size between N2 and HI is unlikely to reflect differences in the size of interphase nuclei. When we measured the diameters of nuclei of embryos between 4 and 8 cell stages, contrary to what we might expect from the differences in step size and scaling coefficient, N2 nuclei were slightly smaller on average compared to HI (5µm compared to 5.15µm; Additional File 1: Figure S2B).

Power-law fitting not only reveals the folding properties of chromosomes, but also serves as a means to normalize the spatial distance measurements by taking into consideration the polymer nature of the chromosomes [23, 26, 44]. Normalization of the N2 and HI spatial distances revealed general similarities in folding complexities between HI and N2 (Fig. 2D). For example, the distances within the left and right arms were smaller than expected by the power-law function for both N2 and HI, suggesting these regions were highly folded. In addition, the distances between the center and the right arm were larger than expected, consistent with a barbell configuration for both strains, by population average analysis [26]. Despite the overall similarity between both strains, they showed some differences in pairwise distances, as revealed by the normalized significant changes between N2 and HI (Fig. 2E).

Previously, we observed that individual chromosomes in vivo assumed conformations that were distinct from that of the population average [26]. The prevalent folding patterns can be revealed by unbiased clustering, which we undertook here. To do so, we pooled N2 and HI, adjusted for the difference in chromosome sizes and performed clustering on the mix (Fig. 2H, “Methods”). Co-clustering of pooled N2 and HI traces ensured consistency and comparability between the data for statistical purposes. We used a chi-square test for independence to compare the distribution of traces into clusters. Chi-square statistics assess the association between the type of chromosome (N2 vs HI) and clusters, while Cramer’s V indicates the strength of the association while accounting for the influence of large sample size.

We found similar clusters of traces for each strain (Fig. 2F). These consisted of chromosomes with one arm highly folded, the other arm folded, chromosomes with both arms folded or distended chromosomes with little folding. These resemble the configurations seen previously for homozygous N2 [25]. The distribution between clusters was comparable, even though it was statistically significant (*p*-value = 5.03 × 10^−6^), likely because of the very large number of traces analyzed. However, the magnitude of this association was very small, as measured by Cramér’s V (0.08 where 0 represents independence and 1 complete association). We conclude that N2 and HI ChrV show an overall similar structure to each other at the Megabase scale.

### A new pipeline to analyze N2:HI hybrids

**Fig. 3:**
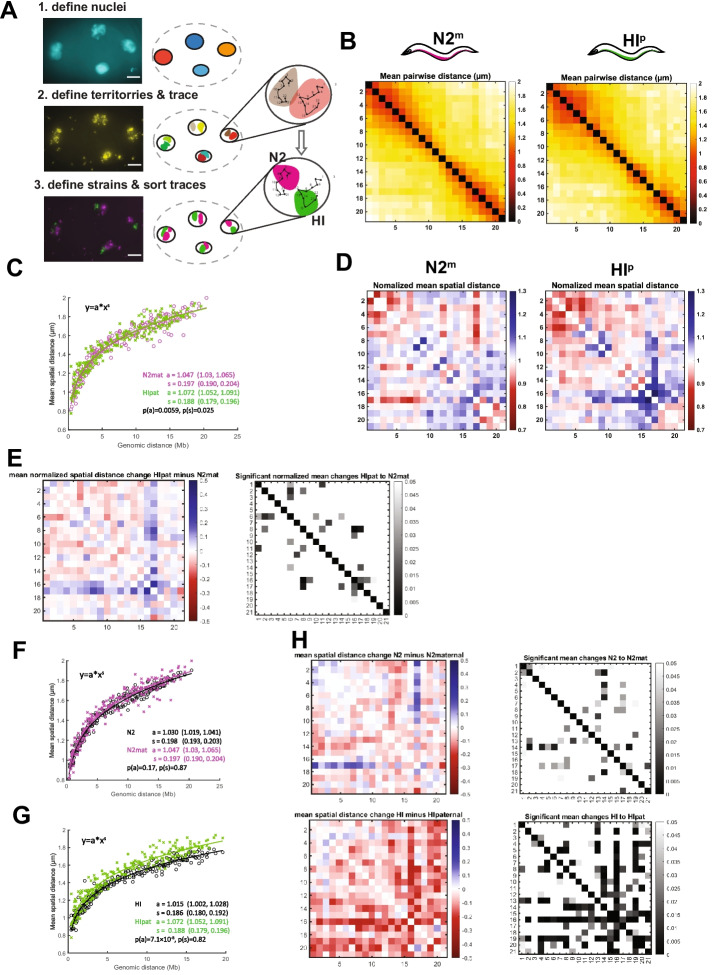
HI paternal chromosomes decompact when subjected to the N2 maternal environment. **A** Example of Z-projections of raw data collected during haploid-specific chromosome tracing and schematic representation of image segmentation, tracing and sorting of traces. Scale bar, 5µm. **B** ChrV mean distance matrix for traces derived from hybrid embryos (2–40cells) from crosses between N2 hermaphrodites and HI males, N2 maternal traces (left) and HI paternal traces (right), colored by distance (in μm). *N* = 1384 (N2^m^) & *N* = 1066 (HI^p^). Axis numbering represents positions across the chromosome as shown in Figure 1C. **C** Power-law fits of mean pairwise distance for N2^m^ data (magenta) and HI^p^ data (green). s = scaling exponent of spatial distance, a = step size. Values in brackets are 95% confidence intervals of the fit. *P*-values are acquired from a linear regression analysis of log-transformed data. **D** Normalized mean spatial distances for N2^m^ and HI^p^ traces shown as observed over expected spatial distances as determined by the power-law fit in **C**. Red denotes regions that are closer together than expected from the fit and blue regions that are further away. **E** Differences in normalized mean spatial distances between N2^m^ and HI^p^ traces (left) and *p*-value (right). Red denotes regions that are closer together than expected from the fit and blue regions that are further away. **F** Power-law fits of mean pairwise distance for N2^m^ data (magenta) and N2 homozygous data (black). s = scaling exponent of spatial distance, a = step size. Values in brackets are 95% confidence intervals of the fit. *P*-values are acquired from a linear regression analysis of log-transformed data. **G** Power-law fits of mean pairwise distance for HI^p^ data (green) and HI homozygous data (black). s = scaling exponent of spatial distance, a = step size. Values in brackets are 95% confidence intervals of the fit. *P*-values are acquired from a linear regression analysis of log-transformed data. **H** Differences of mean pairwise distances in µm (left), and *p*-value (right) of N2^m^ and N2 homozygous (top) and HI^p^ and HI homozygous (bottom). Red marks regions that are closer together than expected from the fit and blue regions that are further away. Axis numbering in **B**, **D**, **E**, and **H** represents positions across the chromosome as shown in Fig. 1C for all panels

Our next goal was to examine N2 and HI chromosomes after interbreeding. First, we developed and implemented a new image segmentation and tracing pipeline. Like previous chromosome tracing [26], we applied watershed segmentation on the nuclear signal (DAPI staining) to restrict the definition of chromosome territories to the nuclear volumes and remove any background signal from outside the nuclei (Fig. 3A, step 1). Watershed segmentation is a method of image processing that automatically separates individual elements in the foreground (e.g., nuclei) from the background (e.g., cytoplasm). We implemented watershed segmentation using MATLAB as performed previously [26] (https://www.mathworks.com/help/images/marker-controlled-watershed-segmentation.html).

Next, watershed segmentation was applied to images of chromosome territories. This step defined the volumes in which chromosomes were traced using a nearest neighbor approach (Fig. 3A, step 2; [26]). The underlying assumption of this approach is that region “*n*” along the chromosome connects to the closest focus in 3D space that was detected for region “*n*+1,” and not a more distant “*n*+1” focus, which we assume belongs to another chromosome. This approach agrees well with simulated polymer models [45]; however, there is no absolute way to assess what the “real” chromosome path is, and therefore it is not possible to calculate an error rate.

The strain-marking territories were segmented for N2 and HI, and the resulting volumes overlayed with the traces generated in the previous step (Fig. 3A, step 3). Since the primary ChrV probes did not overlap perfectly with the strain-marking probes (Fig. 1G), we classified traces into N2 or HI based on whether the majority of regions of traces were located within or closest to a strain-marking volume for N2 or HI. Therefore, we calculated the smallest Euclidian distance for each region within a trace to the boundary points of the strain-marking volumes for N2 and HI. If the region was based on this calculation closer to N2, it was classified as N2 and vice versa for HI. To account for wrongfully segmented and traced chromosomes in the high-throughput analysis, we added several drop-out criteria for traces, which were excluded from further analysis: (i) the presence of more than 4 traces in one nucleus or (ii) the presence of more than 2 traces per strain per nucleus. These situations are biologically impossible in a wild-type setting and likely reflect over segmentation that has split a territory (chromosome) into two.

### The paternal chromosome mimics the maternal conformation in N2^m^ x HI^p^ hybrids

We used our new tracing pipeline on embryos derived from crosses between N2 hermaphrodites (N2^m^) and HI males (HI^p^). The overall conformation of ChrV in these hybrids agreed well with the homozygous conformations, with compacted arms and more open centers (Fig. 3B). Power-law fitting revealed that both chromosomes showed very similar genomic distances vs. spatial distance relationships with each other, and these resembled N2 homozygotes (Fig. 3C,F,G). Only a minority of pairwise distance changes were significant between N2 maternal (N2^m^) and HI paternal (HI^p^) chromosomes, indicating that they were highly similar overall (Fig. 3E).

Despite the similarities, power-law fitting revealed that the N2^m^and HI^p^ chromosomes differed slightly in step size (1.05 vs. 1.07) and scaling exponent (0.198 vs. 0.186). The scaling exponents were unchanged for HI and N2 chromosomes with respect to their homozygous conformations, but the step size for HI^p^ chromosomes increased, from 1.015 in homozygotes to 1.072 in the N2^m^ background, whereas it remained virtually unchanged for N2^m^ compared to N2 homozygotes. Thus, the HI^p^ chromosome became more similar to the N2 chromosome with regard to step size. Closer examination of mean pairwise distance changes of N2^m^ and HI^p^ chromosomes (compared to homozygous chromosomes from un-crossed embryos), revealed that substantially more regions changed in HI than in N2. Almost all these regions decompacted in HI^p^ chromosomes (red, Fig. 3H). Statistically, N2 maternal (N2^m^) showed no statistical difference with N2 homozygotes, while the step size of HI paternal (HI^p^) was statistically different from HI homozygotes. This result reveals that the HI^p^ chromosome decompacts when subjected to the N2^m^ environment and implies that the paternal chromosome was influenced by the maternal environment.

**Fig. 4:**
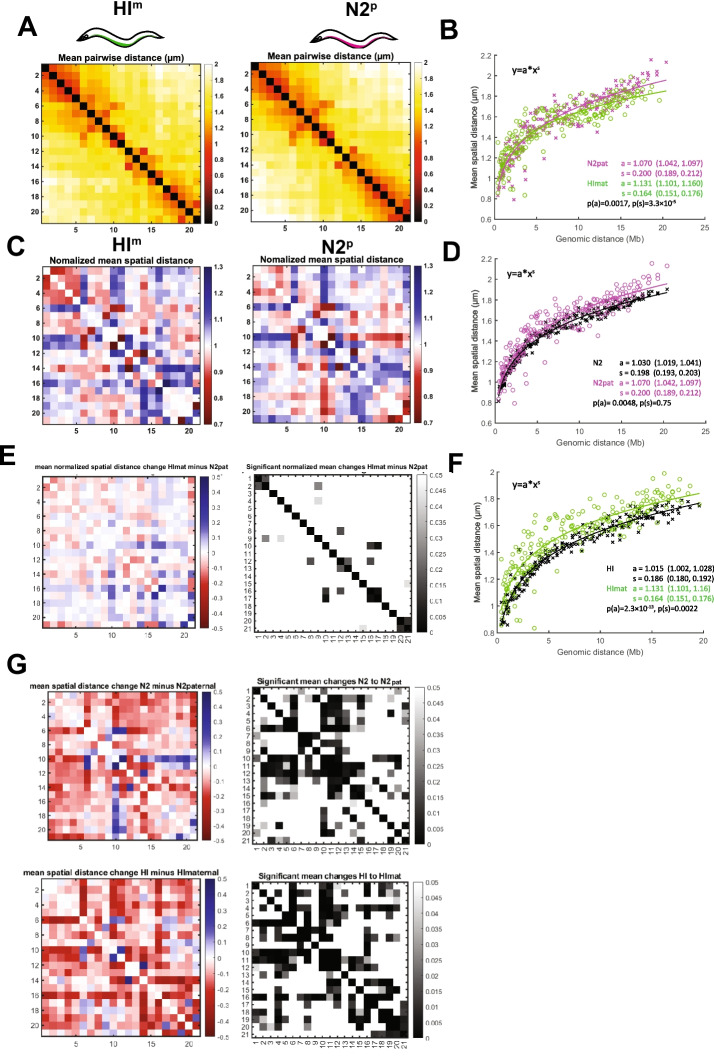
N2 chromosomes influence HI chromosomes in trans. **A** ChrV mean distance matrices for traces derived from hybrid embryos (2–40cells) from crosses between HI hermaphrodites and N2 males, HI maternal traces (left), and N2 paternal traces (right), colored by distance (in μm). *N* = 1125 (HI^m^) & *N* = 1254 (N2^p^). Axis numbering represents positions across the chromosome as shown in Figure 1C. **B** Power-law fits of mean pairwise distance for N2^p^ data (magenta) and HI^m^ data (green). s = scaling exponent of spatial distance, a = step size. Values in brackets are 95% confidence intervals of the fit. *P*-values are acquired from a linear regression analysis of log-transformed data. **C** Normalized mean spatial distances for N2^p^ and HI^m^ traces shown as observed over expected spatial distances as determined by the power-law fit in **B**. Red denotes regions that are closer together than expected from the fit and blue regions that are further away. **D** Power-law fits of mean pairwise distance for N2^p^ data (magenta) and N2 homozygous data (black). s = scaling exponent of spatial distance, a = step size. Values in brackets reflect the 95% confidence intervals of the fit. *P*-values were acquired from a linear regression analysis of log-transformed data. **E** Differences in normalized mean spatial distances between N2^p^ and HI^m^ traces (left) and *p*-value (right). Red marks regions that are closer together than expected from the fit and blue regions that are further away. **F** Power-law fitting of mean pairwise distance for HI^m^ data (green) and HI homozygous data (black). s = scaling exponent of spatial distance, a = step size. Values in brackets are 95% confidence intervals of the fit. *P*-values are acquired from a linear regression analysis of log-transformed data. **G** Differences of mean pairwise distances in µm (left), and significant mean changes (right) of N2^p^ and N2 homozygous (top) and HI^m^ and HI homozygous (bottom). Red marks regions that are closer together than expected from the fit and blue regions that are further away. Axis numbering in **A**, **C**, **E**, and **G** represents positions across the chromosome as shown in Fig. 1C for all panels. Axis numbering represents positions across the chromosome as depicted in Fig. 1C

To test the influence of the maternal HI (HI^m^) environment, we performed the reciprocal cross between HI hermaphrodites and N2 males. Again, we found that the overall conformation agreed well with HI and N2 homozygotes, with compacted arms and more open centers (Fig. 4A, C). Power-law fitting showed differences between N2 paternal (N2^p^) and HI^m^ chromosomes in the scaling exponent (0.200 vs. 0.164) but the N2 value resembled the homozygous N2. As before, only a minority of N2^p^ and HI^m^ chromosomes’ pairwise distance changes were significant (Fig. 4E). This result confirms that HI and N2 chromosomes behave similarly within hybrids.

Despite the similarities, the values indicate that the paternal (N2) chromosome decompacted compared to the homozygous (N2) chromosome as indicated by the increased step size (1.07 vs. 1.03; Fig. 4D). The HI^m^ chromosome changed in two ways compared to the HI homozygous chromosome, first by a decreased scaling exponent (from 0.19 to 0.16) and second by an increased step size, and the result was highly significant (1.015 to 1.131; Fig. 4F). This result suggests that each chromosome was subtly changed in this particular hybrid, with the N2 influencing HI. When we compared mean pairwise distance changes of HI^m^ and N2^p^ chromosomes with their homozygous counterparts, we detected significant changes for both chromosomes. Consistent with the values from power-law fitting, most regions became decompacted (Fig. 4G).

Taken together these data suggest that paternal chromosomes are influenced by the maternal environment in both crosses. Since we also find that HI^m^ chromosomes change in the presence of N2^p^, we hypothesize that the N2 chromosomes also influence HI chromosomes in trans, while N2 chromosome structure seems to be more resistant to influences by the HI chromosome.

### Cluster analysis reveals a new domain in HI^m^ x N2^p^ crosses

**Fig. 5:**
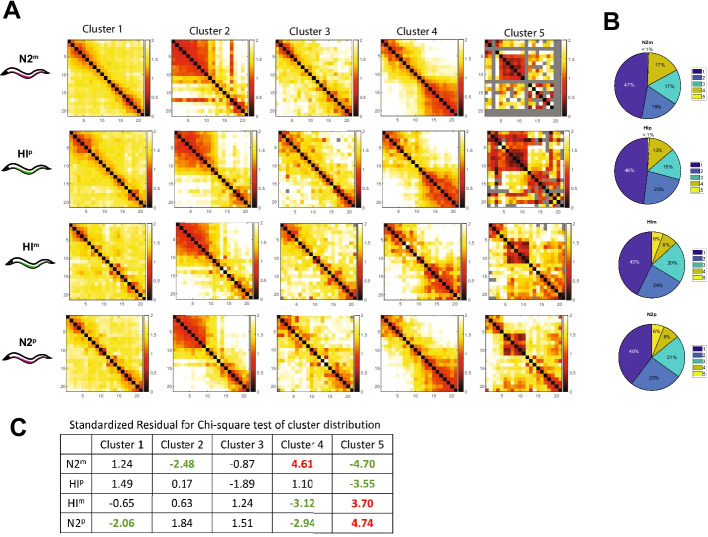
HI^m^ and N2^p^ chromosomes show altered large-scale folding. **A** Matrices of mean pairwise distances in µm for subpopulations of chromosome conformations for crosses as determined by unsupervised clustering. Traces from both crosses were pooled and analyzed together as described in Figure 2H. Missing data are marked in gray. Axis numbering represents positions across the chromosome as shown in Figure 1C. **B** Distribution of traces within clusters from A shows all crossed strains have similar cluster proportions, except for cluster 5 which is enriched in HI^m^ and N2^p^ traces. Chi-square statistic was 138.209, *p*-value= 0 and Cramér’s *V*=0.120. **C** Standardized residual for chi-square testing to reveal conformations that are enriched (red) or deficient (green) relative to expectation

We performed unbiased cluster analysis on the mated strains. To enable statistical comparisons, we pooled the chromosome traces together and examined their clustering behavior for all crossed animals (HI^m^, N2^p^, N2^m^, HI^p^). In this experiment, we detected the prevalent folding patterns seen before, but the enrichment varied depending on the cross. The most noticeable difference was observed for the HI^m^ x N2^p^ cross, where Cluster 5 contained a small, compact structure or domain located approximately 6–12 Mb along the chromosome, with sharp boundaries relative to neighboring sequences. This cluster constituted 6% of the total in the HI^m^ x N2^p^ cross, but <1% in the reciprocal N2^m^ x HI^p^ cross, and was not observed in clusters of the original N2 or HI homozygous strains even when finely resolved to 11 clusters (Figure 2, data not shown, [26]). We calculated the standardized residuals for HI^m^ and N2^p^ as 3.70 and 4.74 respectively, indicating a strong enrichment (Fig. 5C). Conversely, the values for N2^m^ and HI^p^ were −4.70 and −3.55 indicating a deficiency of this configuration (Fig. 5C). Thus, crosses can engender or enrich for new chromosome configurations. The HI^m^ x N2^p^ cross also exhibited a reduction in Cluster 4, with large-scale looping along the right arm. The most prevalent cluster was an extended chromosome, seen in all four chromosomes (N2^m^, N2^p^, HI^m^, HI^p^). These data suggest that while the cross of HI^p^ males with N2^m^ mothers preserves the expected categories, the reciprocal cross altered large-scale folding along the chromosome. Furthermore, the differences in clustering between crosses is in line with increased differences seen in mean pairwise distance changes in crosses of HI mothers with N2 males (Figure 4).

### Homologous chromosomes do not align

Transcriptional regulation depends on cis-regulatory sequences that are typically adjacent to target promoters [27, 46]. In *Drosophila* and other Dipterans, homologous chromosomes are paired in interphase somatic cells, allowing for interchromosomal interactions between enhancers and promoters, a phenomenon termed transvection [47]. Sequences that promote transvection stabilize the association between alleles, leading to a significant proportion of aligned homologs within 300–400nm of one another (e.g., 20% for *gypsy*) [48]. Methods to study physical interactions of homologous chromosomes are limited since the distinction between homologous chromosomes is not always possible [29].

**Fig. 6:**
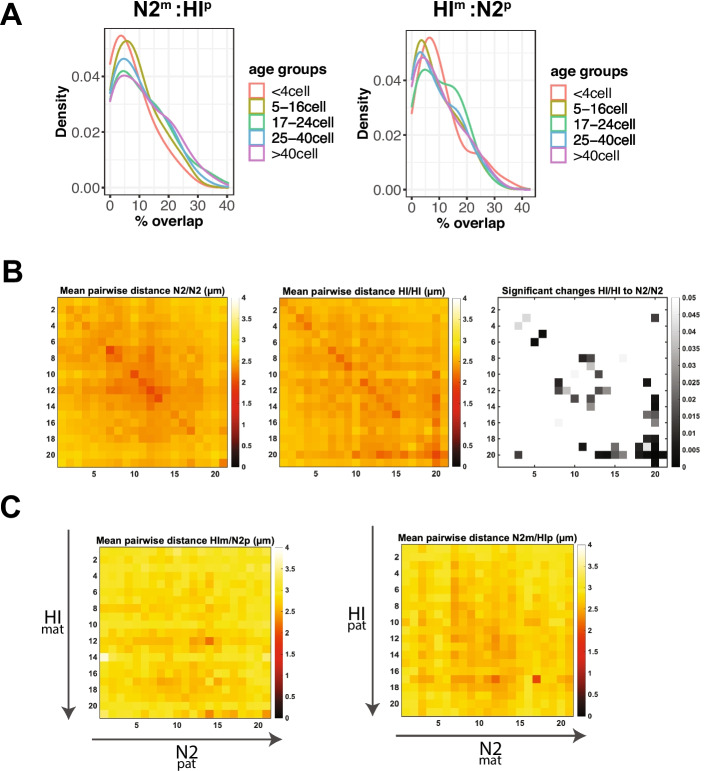
Homologs frequently intermingle, but rarely align. **A** The proportion of chromosome pairs (“Density” *y* axis) is graphed with respect to the degree of territory overlap (% overlap), calculated as the fraction of voxels in the overlap region divided by the total voxels for both territories (*x* axis). Territory regions were calculated from the N2 and HI probes. N2 and HI chromosomes from crosses between N2^m^ and HI^p^ in different age groups of embryos indicated by color-coding. The % overlap is defined as the ratio between voxels of overlapping N2 and HI marker signal, and total number of voxels defining the N2 territory and HI territory. **B** Mean spatial distances between all regions of one homolog against all regions of the other homolog for N2 (left) and HI (middle) ChrV in homozygous embryos, colored by distance (in µm). **C** Mean spatial distances between all regions of one homolog to all regions of the other homolog of HI^m^ and N2^p^ chromosomes (left) and of HI^p^ and N2^m^ (right), colored by distance (in µm). Axis numbering in **B** and **C** represents positions across the chromosome as shown in Figure 1C

To determine the degree of overlap between homologs within single nuclei, we used stringent image segmentation on Z-stacks of the N2 and HI markers acquired during tracing of N2^m^/HI^p^ and HI^m^/N2^p^ hybrid embryos. We defined the territory volume the N2 and HI ChrV occupied as the number of voxels which contained a signal for N2 or HI. The overlap was then calculated as the number of voxels which were marked by both N2 and HI divided by the total number of voxels occupied by the total N2 and HI territories (Fig. 6A). Since we detected a small background staining in N2 embryos with the probes that were designed to mark the HI strain (Fig. 1E), we used a cut-off of 5%. Below this value, we considered the homologs non-overlapping. We found that in HIm/N2p embryos 37% of nuclei showed no overlap, and of the remaining nuclei, 26% had an overlap larger than 15% of the strain-marking volume, while in N2m/HIp embryos 32% of nuclei showed no overlap and of the remaining nuclei, 30% had an overlap larger than 15% of the strain-marking volume. Thus, homologs display frequent territory overlap in *C. elegans* embryos.

We asked if the overlap between homologs was related to developmental stages (Fig. 6A). A Kolmogorov-Smirnov test was performed to test the difference in overlap ratio between embryo developmental stages (Additional File 1: Figure S4). Data of <4 cell was merged with 5–16 cell to reach the sample size requirement for the test. *P*-values suggested that there was a significant deviation in overlap ratio between 0 and 16 cell embryos and later ages in N2xHI and 17-24 cell embryos and other ages in HIxN2 (Additional File 1: Figure S4 C, F). However, because the actual difference indicated by K-S statistics and difference in mean ratio (Additional File 1: Figure S4 A, B, D, E) was small, there was no statistical support for a biologically relevant difference in overlap ratio between different development stages. Deeper analysis of the 1–4 cell stages could reveal if the reduced overlap is real.

We next asked if certain regions along homologs overlapped preferentially. Chromosome tracing records the trajectory and hence the location of individual regions of single chromosomes within single nuclei. To determine whether individual regions of one homolog were preferentially closer to specific regions on the other homolog, we first focused on traces derived from homozygous N2 and HI embryos, respectively. We filtered our data to restrict the analysis to all nuclei that contained exactly two traces and therefore had not yet undergone discernable DNA replication. We measured the distances of all regions along one chromosome to all regions along the homologous partner chromosome within the same nucleus for N2 and HI homozygous embryos (Fig. 6B). Distances were generally fairly large, on the order of 2.5 µm in nuclei that were on average 5.3 µm in diameter (Additional File 1: Figure S2). While this absolute distance may be skewed by fixation conditions, this result suggests that although homologous chromosomes frequently overlap a portion of their territories, homologous genes are not more closely aligned with one another than with other points along the chromosome. We also note that there were few significant differences for chromosomes from N2 vs HI (Fig. 6B).

We also examined our hybrids and measured pairwise distances between all regions along HI^m^ and N2^p^ chromosomes, and N2^m^ and HI^p^. We restricted the analysis to all nuclei that contained exactly two traces. The data revealed that *C. elegans* does not stably align homologous domains and any inter-homolog interactions might, similar to mammalian genomes, be confined to particular regions of the chromosome or occurring within specific cell types [49]. Interestingly, we also observed that distances between HI^m^/N2^p^ chromosomes were typically larger than those for N2^m^/HI^p^, perhaps reflecting the larger size of HI nuclei (Additional File 1: Figure S2B).

## Discussion

### Chromosome tracing for homologous chromosomes

This study analyzes chromosome conformations from maternal and paternal genomes using crosses between divergent *C. elegans* strains and chromosome tracing. We have developed probes and an analysis pipeline to distinguish between N2 and HI strains after FISH, and we have used these tools to determine the conformations of maternal vs. paternal ChrV at the Megabase scale. Previous studies have distinguished chromosomes using DNA FISH in other species [50], but to our knowledge this is the first adaptation of chromosome tracing to distinguish maternal vs. paternal chromosome conformations during development. We note that our approach could be adapted for other purposes. For example, combining smFISH with strain-specific, territory probes would enable analysis of gene transcription from the maternal vs paternal chromosome, even in the absence of SNPs within the gene of interest.

The chromosome tracing method has pros and cons compared to other, biochemical approaches. It uniquely allows direct visualization of many genomic regions in cells without disrupting the nuclear environment, and direct quantitative measurement of chromosome conformation without the need for DNA ligation [31]. The major limitation of tracing is that, compared to sequencing-based biochemical approaches like Hi-C, tracing technology does not yet reach very high resolution while maintaining genome-wide capability. However, another strength is that chromosome tracing is also a single-molecule approach, which enables researchers to compare the copies of the same chromosome in each nucleus. Previous tracing studies have not been able to distinguish the parent-of-origin of each chromosome [26, 51–55], and this study provides this advance.

Studies of haploid-resolved chromosome conformations have been largely limited to Hi-C methods, which tend to rely on population averaging. Our tracing and territory strain-marking approach enabled us to determine the conformation of many regions along single chromosomes, including regions lacking SNPs or indels. In this first study, the strain-marking library relied on insertions larger than 1000nts. Future libraries could increase the density of probes by including sequences from smaller insertions. This adaptation would in turn allow the discrimination of less divergent strains or, in the case of tracing libraries, for subregions of chromosomes, the marking of chromosomes and regions which are less divergent. The approach we presented therefore is adaptable for usage in a variety of conditions. In addition, chromosome tracing by multiplexed FISH in general has the added benefit of preserving the spatial properties of the region under study within the nucleus, such as proximity to the nuclear periphery. It also preserves the tissue in study, making it possible to relate chromosome traces to the presence of cellular features outside of the nuclei [23, 26, 52, 56].

### N2 and HI conformations

Our data reveal that (i) N2 and HI embryos have similar chromosome conformations, with a few differences in particular regions; (ii) after inter-strain crosses, HI chromosomes adopt the configuration of the N2 maternal configuration, implicating the maternal environment for influencing chromosome morphology; (iii) HI mothers crossed to N2 fathers generate a low number of chromosomes with a novel configuration. It is unclear if this configuration serves a purpose, but we note that it was not detected in other genetic backgrounds; (iv) homologous chromosomes frequently intermingle their territories; however, homologous alleles rarely align.

We found that the chromosome conformations of HI and N2 were similar and resembled barbells, as seen previously for N2 homozygotes [26]. This was true for both self-progeny and cross-progeny between strains. A minority of pairwise distances along ChrV showed significant differences in inter-probe distances, even though ChrV is the most divergent of chromosomes between the strains. Long-read sequencing had previously identified several large rearrangements, one of which translocated 170kb of N2 ChrV left arm to the left arm of ChrII in CB4856 (HI) [38]. None of these rearrangements or SNPs influenced the larger-scale chromosome organization of ChrV. The clusters of subpopulations between the two strains were similar as well. We observed two clusters with one of the two chromosome arms being compacted by long-range folding, and two clusters with more uniform compaction along the entire chromosome. Despite their average similarity, HI clusters showed smaller distances in left-right-arm measurements compared to N2, suggesting the inter-arm proximity may account for the higher compaction of HI ChrV compared to N2 ChrV.

Using our haploid chromosome tracing method, we found that paternal traces are overall less compacted than the homozygous population of traces of the same strain. One hypothesis is that paternal traces are subjected to a bigger influence by the maternal environment. Comparisons of our traces showed few regional differences, which might reflect the robustness of the larger-scale chromosome organization. We note that the resolution of the library was not high enough to detect smaller, regional differences between alleles. Therefore, we cannot exclude that maternal and paternal chromosomes are different on the local chromatin level. Future studies with smaller-scaled chromosome tracing libraries are needed to tackle this question.

What might account for sex-specific differences? Chromatin bearing modified histones is inherited from *C. elegans* parents, including marks associated with germline transcription such as histone H3K4me2, histone H3K36me3, and histone H3.3, as well as marks associated with germline silencing, such as histone HTAS-1, H3K9me3, and H3K27me3 [9, 10, 57–61]. It is possible that differential modification of maternally vs. paternally donated chromosomes could account for differential compaction in descendants. For example, H3K9me3 and H3K27me3 are associated with chromatin compaction and are enriched on the X chromosome in the male germ line and early embryo [9]. Conversely, it is possible that organization of the genome in sperm or oocytes influences gene expression in the progeny beyond local chromatin marks. This feature may extend beyond the X chromosome, but it is currently unknown whether autosomes also show parent-of-origin differences. The N2/HI system described here can help resolve this issue in future studies, by providing a means to distinguish chromosomes from each parent.

In *Drosophila* and other Dipterans, homologous chromosomes are paired in somatic cells [47] and, for example, 20% of alleles show pairing for genes undergoing transvection (e.g., *gypsy* [48]). Strict pairing of homologs in other organisms has not been widely observed. In mammals, pairing has been seen for sister chromatids after replication [45], but pairing of homologs is rare, tissue-specific, and restricted to particular regions of chromosomes [49]. Earlier studies in mice using FISH demonstrated that chromosomes tend to locate in heterologous neighborhoods [62–64], and work in human cells found larger distances between homologs than heterologs [65]. These observations imply that inter-homolog interactions may be rare beyond dipterans. In this study, we found that in *C. elegans* homologs of ChrV frequently intermingle, but homologous regions rarely align closely. Given the large number of chromosomes sampled (in the thousands), 10 or 20% paired chromosomes would have been detected.

## Conclusions

This work establishes a method and analysis pipeline to track the parent-of-origin of *C. elegans* chromosomes. Using this approach, we find chromosomes adjust their folding parameters in distinct parental environments, but these effects are slight. We also observed robust intermingling between territories of homologous chromosomes; however, homologous alleles rarely aligned. The data suggest that HI x N2 crosses will facilitate studies of intergenerational chromosome architecture.

## Materials and methods

### Strain maintenance and crossing experiments

Bristol (N2) and Hawai’ian (CB4856) strains were maintained at 20°C and grown on OP50 [66]. For crossing experiments, the evening before embryo collection, mid/late L4 hermaphrodites were placed together with equal number of L4 or young adult males of the opposite strain on a mating plate (regular 6-cm plate with agar cut to ~1/6 size).

### Probe design and synthesis

#### Strain-specific probes set

Pools of oligo probes were generated from sequences that encompassed insertions > 1000nts for ChrV of Bristol (N2) and Hawai’ian HI (CB4856; [32]). For each probe region, we extracted the genomic sequence of the *C. elegans* genome assembly (Ce10 for Bristol and Thompson for HI). The probes were predicted by OligoArray2.1 [39], which uses NCBI BLAST 2.2.26 and the OligoArrayAux secondary structure predictor. The parameters used were as follows: melting temperature 60–100°C, no cross-hybridization or predicted secondary structure with a melting temperature greater than 70°C, GC content 30–90%, no stretches of 7 or more identical nucleotides. OligoArray2.1 was run on the SCICORE high performance computing cluster at the University of Basel.

Next, the predicted 30-nt probes were checked for unique sequence binding to the genome of interest using NCBI BLAST 2.9.0+ (Ce10 for N2-specific probes and Thompson-genome for HI-specific probes). In addition, all probe sequences were confirmed to not bind to the other genome by NCBI BLAST 2.9.0+ (Thompson for N2-specific probes and Ce10 for HI-specific probes). Probes sequences were then fused to tail sequences that included primer binding sites for amplification and secondary oligo binding sites unique for either the entire N2-specific or the HI-specific probe sets.

For N2-specific probes after detecting a slight signal when hybridizing to HI embryos (Fig. 1E), we used NCBI BLAST 2.9.0+ to blast the N2-specific probe library against the HI genome released by Kim et al., which was performed using long-read sequencing. We found 536 of the 5577 N2-specific probes for ChrV binding along the HI ChrV. Two hundred fifty-three of which were located very close to each other within 9.5kb between 17.32 and 17.33Mb along ChrV of the Kim genome. We think that this concentration of probes results in the background staining in HI.

#### Shared chromosome tracing probes

Chromosome tracing probes for ChrV were as described previously, with 22 100-kb regions located in the center of previously identified TADs [26]. Due to low signal to noise ratio for one of the 22 regions of this tracing library, we excluded it from our experiments (TAD11).

#### Probe amplification

The probe libraries were amplified and labeled with fluorophores (whole-chromosome library only) [23, 25, 26]. In brief, probes were amplified using limited cycle PCR (Phusion Hot Start Master Mix, Life Technologies), high yield in vitro transcription (HiScribe T7 Quick High Yield RNA Synthesis Kit, NEB), and cDNA synthesis reactions (Maxima H Minus Reverse Transcriptase, Fisher Scientific). Only for the whole-chromosome library (ChrV), probes were 5′ labeled with a fluorophore (ATTO 565 (IDT)) during the reverse transcription step. Probes were purified using the DNA Clean & Concentrator kit with Spin IC columns after PCR, and Spin V columns using Oligo Binding Buffer after cDNA synthesis (all reagents from Zymo). All probe sequences and primers used in this study are available in Additional File 2: Table S1-S3.

### Embryo sample preparation and in situ hybridization

Embryos were dissected, fixed, and hybridized to DNA FISH probes as described previously [26]. In brief, embryos from young gravid adults were dissected in ddH2O and transferred to a round coverslip (Bioptechs) coated with Poly-L-Lysine (Sigma). Embryos were fixed with 1% paraformaldehyde/0.05% Triton X-100, frozen on dry ice, freeze cracked and submerged in ice-cold Methanol for 5 minutes [25, 67]. After washing once with 1× PBS and three times with 1×PBS/0.5% Triton, samples were treated with RNaseA (0.05mg/ml) for 30min at 37°C and blocked using hybridization buffer (10% dextran sulfate / 0.1% Tween-20/ 2X SSC/ 50% formamide) for 1h at 37°C. Primary probes for the whole-chromosome library (ChrV) and strain-specific probe sets were diluted to ∼1μM each in hybridization buffer. Probes were annealed at 80°C on a hot metal plate for 10min and hybridized for at least 16h at 37°C. Slides were first washed with 2× SSC/50% formamide for 1h at 37°C and washed additionally 2 times using 2× SCC and 2 times 0.5× SCC using pre-warmed buffers. Slides were then stored at 4°C or imaged immediately.

### DAPI staining and microscopy chamber assembly

Nuclear staining and flow chamber assembly were performed as described previously [25]. Fixed embryos on round cover slips were stained with DAPI in 2× SCC (1:1000, Thermo Fisher) for 5–10min immediately prior to imaging and washed 3 times with 2× SCC for 5–10min.

Specimens were mounted using a flow cell (Focht Chamber System 2 (FCS2®), Bioptechs), equipped with a micro aqueduct and attached to a home-build fluidic system [68] attached to the microscope. During flow cell assembly, 0.1µm Tetraspeck (ThermoFisher) beads in 2× SCC were allowed to adhere to the coverslip in order to track sample drift during image acquisition.

### Image acquisition

Images were taken on a Nikon Ti2 equipped with a Photometrics Prime 95B camera, Lumencor SpectraX light source, and Omicron lasers for bleaching the primary probe signals.

Sequential hybridizations, image acquisition, and bleaching of probe signals were operated by an automated program implemented in the NIS Elements Software as described previously [26]. At the start of an acquisition, embryos were selected by strong primary probe signal. For each FOV, the first round of imaging consisted of primary probe imaging using 561nm illumination, nuclear stain imaging using 405nm illumination, and fiducial bead imaging using 488nm illumination, and a total of 30µm were acquired in Z using 200-nm steps for all imaging rounds. The primary probe signal was then bleached to undetectable levels using the Omicron lasers. The following sequential secondary probe imaging steps consistent of 20min incubation of secondary probes (8nM) on stage, washing in 2× SSC/25% ethylene carbonate and imaging using 561nm, 647nm illumination (for probe signals), and 488nm illumination (for bead signals), followed by photobleaching. These steps were repeated for all secondary probe regions sequentially. For acquisition of strain-specific markings, an additional hybridization and imaging step was added, which consisted of 20min incubation of secondary probes (32nM), washing in 2× SCC/25% ethylene carbonate and imaging using 561nm, 647nm, and 488nm illumination. Multicolor 100-nm Tetraspeck beads for 647nm and 560nm illumination were included for later alignment during analysis.

### Generation of chromosome traces

Chromatic aberration was corrected by creating a transformation function in *x*, *y*, and *z* of the 647-nm signal into the 560-nm signal (Wang 2016, Sawh 2020). This function was calculated using images of multicolor 100-nm Tetraspeck beads and aligning beads in the 647 channel to the same beads in the 560 channel. In addition, the beads were visualized after each round of imaging to ensure microscope alignment between rounds of hybridization*.* Foci fitting and assignment was done as published previously [23, 26]. Image segmentation and chromosome tracing was done in MATLAB as described previously with modifications [26]. In brief, for each embryo separately, first nuclei were identified using the DAPI signals. Background signal was subtracted, then noise removed and strong signal smoothened. The image was binarized and the distance transform was computed on the resulting binary image prior to watershed segmentation (https://www.mathworks.com/help/images/marker-controlled-watershed-segmentation.html). This resulted in a volumetric mask of individual nuclei, and we focused on interphase nuclei, based on size and conformation. Next territories were segmented analogous to nuclei segmentation, whereby signal outside of nuclei were excluded using the nuclear mask. This resulted in a volumetric mask, where each volume represented a chromosome. Sister chromatids were not distinguished unless visibly separate dots were visible (i.e., *n*>2 dots per nucleus). Tracing of chromosomes was then performed within the defined chromosome volumes as done previously using the nearest neighbor approach [26]. Nearest neighbor assumptions fit well with both territory domains and, in other studies, with polymer simulations [45]. Within each chromosome trace, the distances between all possible pairs of regions were measured in three dimensions. For each region, we then averaged the observed distance for all traced chromosomes, and this average distance is displayed in the heatmap matrices.

To account for wrongfully segmented and traced chromosomes in the high-throughput analysis, we added several drop-out criteria for traces, which were excluded from further analysis. This included the presence of more than 4 traces (or copies of the chromosome) in one nucleus and the presence of more than 2 traces per strain per nucleus, situations which are biologically impossible in a wild-type setting. These typically arise due to oversegmentation of the territory. We assessed the efficiency of this method to classify traces accurately by counting how often traces were excluded due to these criteria for traces from N2^m^:HI^p^ embryos. Out of the 3299 total traces, 1066 traces were classified as HI and 1384 as N2. The remainder of 849 traces (or 25.7%) were excluded for following reasons: (1) For 108 traces (3.3%), any region was present more than once in the trace, to focus on interphase chromosomes and remove ambiguity about locus positioning. (2) For 232 traces (7%), more than 4 traces were detected in one nucleus. This is likely caused by over segmentation of the primary probe signal, resulting in splitting of a territory. (3) For 88 traces (2.7%), there were equal to or fewer than 4 traces per nucleus, but more than 2 traces were classified as HI. (4) For 261 traces (7.9%), there were equal to or fewer than 4 traces per nucleus, but more than 2 traces were classified as N2. The latter two are presumably caused by inaccurate segmentation of the strain-marking territories. (5) One hundred sixty (4.8%) traces were equally close to the strain-marking territory for N2 and HI and therefore excluded.

Similarly for embryos of crosses between HI hermaphrodites and N2 males, we excluded 654 (21.9%) out of 2982 of all traces. (1) Seventy-eight (2.6%) of traces contained the same regions twice. (2) One hundred twenty-eight (4.3%) traces were excluded because 4 or more traces were detected in one nucleus. (3) For 102 (3.4%) traces, more than 2 traces of HI were present in a nucleus and (4) for 179 (6%) traces more than 2 traces were assigned to N2 within the same nucleus. And (5) 167 (5.6%) traces were excluded because they could not be assigned to either N2 or HI.

### Assignment of traces to N2 or HI in crossing experiments

For experiments where strains were marked using the N2 and HI ChrV library, the strain-marking signals were segmented as done for territories. The generated traces were then classified into HI and N2 traces based on whether the majority of the foci detected were located within or closest to a strain-marking territory. Traces shorter than 4 regions were excluded to prevent misassignments and ambiguous traces, where equal number of N2 and HI was identified, were removed.

### Normalization of distances by power-law fitting and statistical analysis

Spatial distance normalization and statistical analysis of distances was performed as described previously [23, 26].

### Linear regression analysis

Mean spatial distance and genomic distance were log-transformed (Additional file 3: Table S4 lists the genomic coordinates for regions centroids in N2 (Ce10) and HI (Thompson)). And the power-law relationship between the two was transformed into a linear relationship. Different datasets were indicated by a binary group variable.

The regression model was: *Y*=*β*0+*β*1X+*β*2*G*+*β*3(*X*×*G*) +ϵ, with *Y* being the log-transformed mean spatial distance, *X* being the log-transformed genomic distance, *G* being the binary group variable representing the dataset, *X*×*G* being the interaction between *X* and *G*, and *ϵ* being the error term. *β*0, *β*1, *β*2, and *β*3 were the regression coefficients: *β*0 was the intercept for the dataset where *G*=0, *β*1 was the slope of *X* for the dataset where *G*=0, *β*2 represents the difference in intercepts between the two groups, *β*3 represents the difference in the slopes of *X* between the two groups. *β*2 represents the difference in log-transformed intercepts, indicating variations in the coefficient a between the two groups, while *β*3 captures how their power-law exponents *s* differ. The *p*-value of these *β*2 and *β*3 values were used to show the degree of differences between the two groups.

### Pearson correlation between datasets for replicates

For every two replicates, a Pearson correlation coefficient was calculated for mean pairwise spatial distance between regions. MATLAB function *corrplot* was used. For both tracing and statistics, we focused on traces with better hybridization (more regions; >6 probe sets out of 21 total per chromosome and >21 measurements of pairs of regions).

### Cluster analysis

Unsupervised clustering was performed as described previously [26]. Since Region 17 produced fewer datapoints detected in tracing experiments, it was excluded from cluster analysis. Resolutions used were as follows: 0.7 for HI traces, 0.6 for N2 traces, 1.0 for Hi^p^ and N2^m^, 0.7 for Hi^m^, and 0.9 for N2^p^ traces in Seurat. The cluster resolution is an arbitrary value that dictates the number of clusters generated; a large value leads to many clusters, sometimes with similar folding parameters that suggest over-clustering. To avoid over-clustering, we maintained a resolution that generated five clusters (Methods).

### Co-clustering of pooled datasets

For co-clustering, N2 and HI databases were pooled and clustering performed as above. The difference in genomic distance between HI and N2 regions was addressed as follows: a predicted spatial distance for each chromosome was calculated from the genomic distance using the power-law parameters of N2; the measured HI spatial distance was divided by the ratio of HI/N2 calculated distance based on the HI chromosome size and N2 power-law curve. Resolution for co-clustering was 0.7 for HI and N2 pooled homozygotes (Fig. 2) and 0.5 for pooled crossed traces in both directions (Fig. 5).

### Chi-square test for co-clusters

Chi-square tests for independence were performed to assess the difference between the distribution of different types of chromosomes in different clusters. Chi-square statistics was calculated as $\upchi 2=\sum \frac{{({O}_{ij}-{E}_{ij})}^{2}}{{E}_{ij}}$ where ${O}_{ij}$ represents the observed frequency in cell *ij* and ${E}_{ij}$ represent the expected frequency. Given the large sample size, even minor deviations from an independent distribution can result in statistically significant *p*-values. To measure the strength of the association between the types of chromosomes and clusters and to account for the influence of large size, Cramér’s *V* was computed. The formula for Cramér’s *V* is $V=\sqrt{\frac{\upchi 2}{n\times {\text{min}}(k-1,r-1)}}$ where *n* is the total sample size, *k* is the number of clusters, and *r* is the number of types of chromosomes. Cramér’s *V* provides a measure of association that ranges between 0 (indicating no association) and 1 (indicating a perfect association).

To understand which cluster of one specific chromosome type contributed the most to the chi-square statistic, standardized residuals were computed. The standardized residual measured how much the observed frequency deviated from the expectation if the clusters and types of chromosomes were independent from each other. The standardized residual was calculated as ${Standardized\;Residual}_{ij}=\frac{(O_{ij}-E_{ij})}{\sqrt{E_{ij}}}$ where ${O}_{ij}$ represents the observed frequency in cell *ij* and ${E}_{ij}$ represent the expected frequency.

### Analysis of homolog overlap

To calculate the overlap between N2 and HI chromosome territories, stringent image segmentation of chromosome territories as well as strain-marking territories, as described above, was applied on a subset of datasets taken for chromosome tracing of N2^m^xHI^p^ and HI^m^xN2^p^ embryos. Since we generated many datasets and found a high correlation between replicates (Additional File 1: Figure S3C&D), we decided to restrict this analysis to 3 out of 6 datasets for N2^m^xHI^p^ and 2 out of 3 datasets for HI^m^xN2^p^.

The generated 3D masks of N2 and HI markers were overlayed, and the voxels counted where N2 and HI overlapped. The %-overlap was defined as the ratio between overlapping voxels and the total voxel count of N2 and HI marker.

### Intra-homolog distance measurements

Data generated in tracing experiments on N2 homozygous, HI homozygous, and N2^m^xHI^p^ and HI^m^xN2^p^ embryos was used to measure distances between all regions of one homolog to all regions of the other homolog, analogous to inter-chromosome distance measurements. Data was filtered for traces which had exactly one partner trace within the same nucleus so as to restrict the analysis to chromosomes which had not yet undergone replication. Since watershed segmentation occasionally failed to segment nuclei well, partner traces which were found to be further then 6µm away between any region, were exclude, since these most likely resulted from mis-segmentation. The distances of all regions on one homolog to all regions of the other homolog was then calculated on the remaining partner chromosomes.

### Supplementary Information

**Additional file 1:** **Figure S1-S4.**

**Additional file 2:** **Table S1-S3.** Sequences of all probes and primers used in this study.

**Additional file 3:** **Table S4.** Genomic Coordinates of 100-kb Probe Regions for ChrV Whole-Chromosome Tracing in N2 (Ce10) and HI (Thompson).

**Additional file 4.** Review history.

## Data Availability

Raw images of tracing datasets are available upon request from the authors due to large data size. Individual traces derived from tracing experiments are deposited in Zenodo (https://zenodo.org/records/10354806) [72]. Original .nd2 images from figures can be downloaded from Zenodo (https://zenodo.org/records/10356985) [73]. The code generated for this study is deposited on github (https://github.com/gutnsilvi/maternal-vs.-paternal-MultiplexedFISH) [74] and Zenodo (https://zenodo.org/records/10450570) [75]. Some code used in this study was modified from Zhuang and Wang lab protocols [23, 56, 76] as indicated within the code in the repository.
