# Supplementary material for: Multiplex DNA fluorescence in situ hybridization to analyze maternal vs. paternal C. elegans chromosomes
Source: Genome Biol. 2024 Mar 14;25:71. doi: 10.1186/s13059-024-03199-6 (PMC10941459; doi:10.1186/s13059-024-03199-6)
Supplement: Supplementary file 1 — Additional file 1: Figure S1-S4. [file 13059_2024_3199_MOESM1_ESM.pdf]

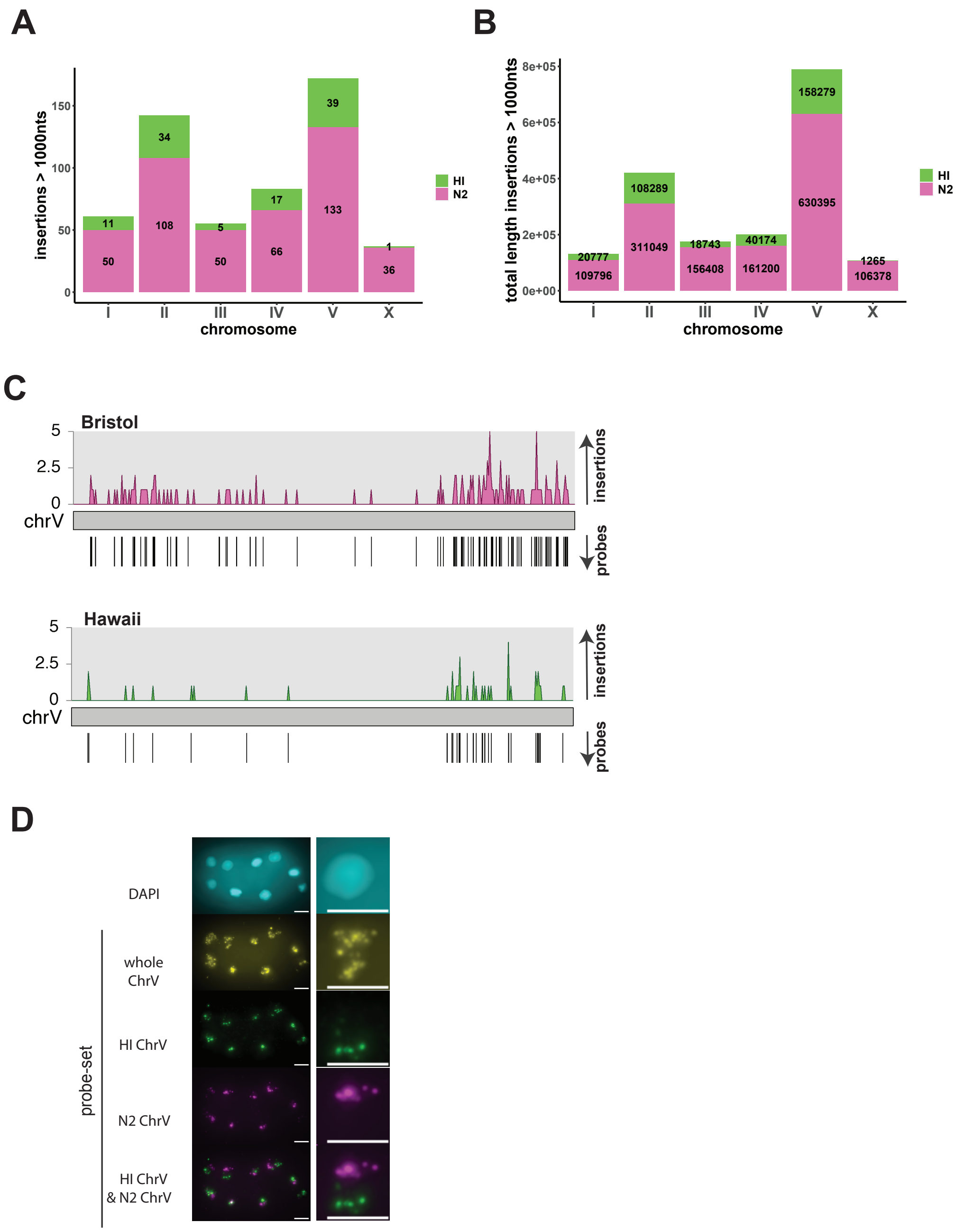

Figure S1: (A) Number of insertions larger then 1000nts present on chromosomes of N2 (magenta) and HI (green).

(B) Total length (in nts) of insertion larger then 1000nts present on chromosomes of N2 (magenta) and HI (green).

(C) Location-density of insertions along N2 (magenta) and HI (green) ChrV binned at 50.000nts (top), and location of final strain-marking probes (bottom).

(D) DNA FISH on embryos derived from crosses between HI hermaphrodites and N2 males, using the whole ChrV library, N2 ChrV and HI ChrV-marking libraries. The haplotypes are well distinguishable. Scale bar, 5µm.

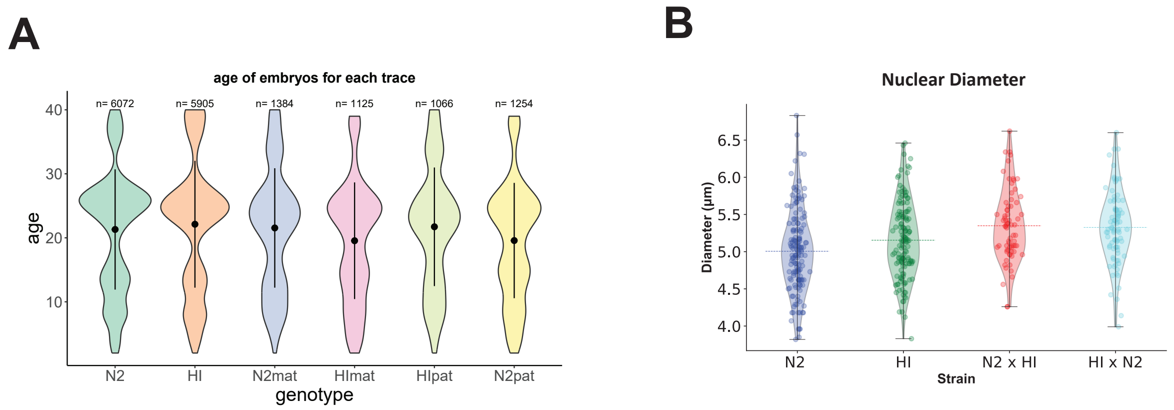

Figure S2

(A) Age of embryos for all traces analyzed.

(B) Nuclear diameters in µm for 4-8 cell stage embryos for N2 and HI, as well as $N2^{m}$ x HI^{p} and HI^{m} x $N2^{p}$ embryos.

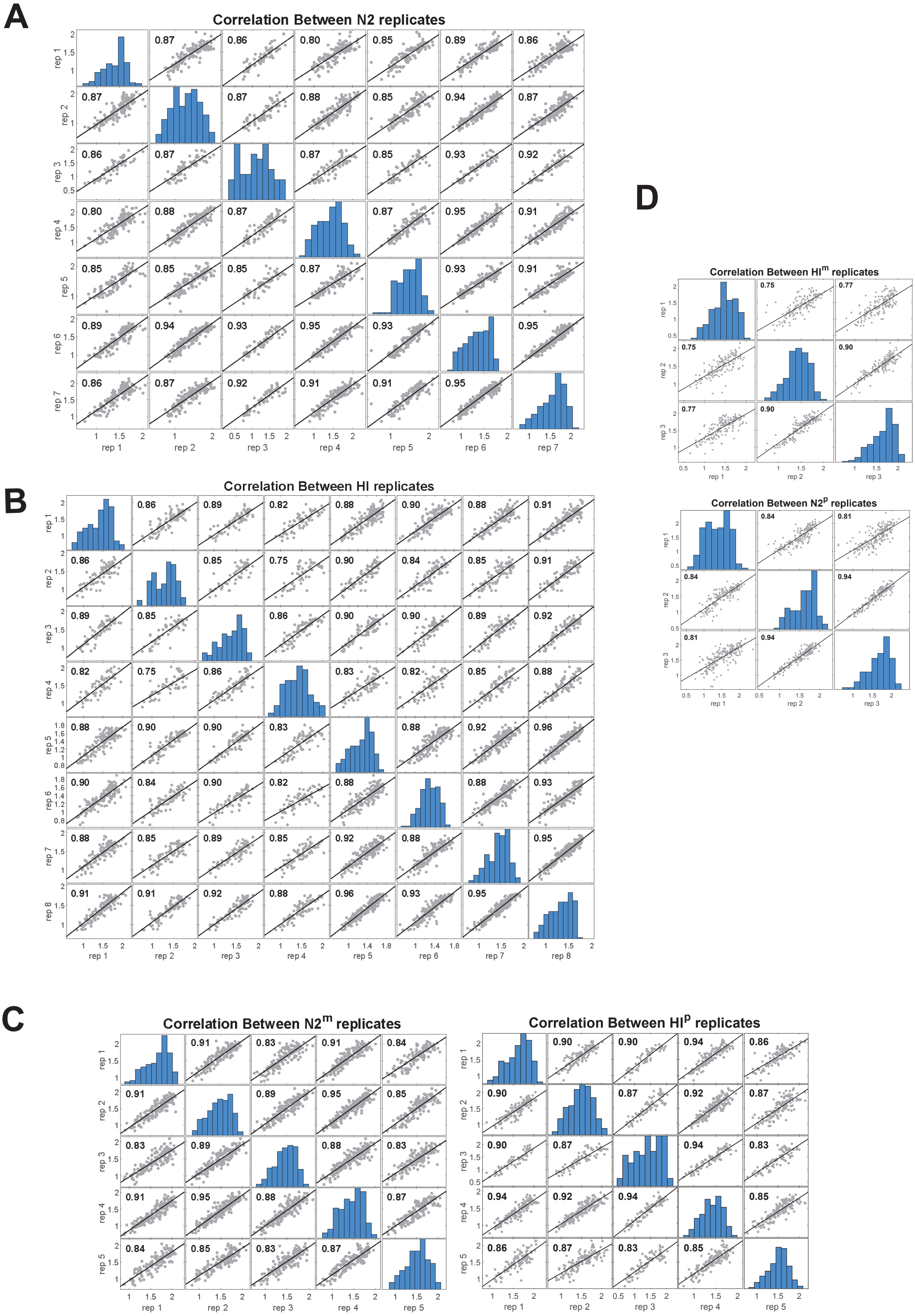
A
Correlation Between N2 replicates
B
Correlation Between HI replicates
C
Correlation Between N2^{m} replicates
Correlation Between HI^{p} replicates
D
Correlation Between HI^{m} replicates
Correlation Between N2^{p} replicates

Figure S3

Pearson's correlation coefficients of mean distance between replicates.

In each off-diagonal subplot is a scatterplot of mean distance of all region pairs for two replicates, with a least-squares reference line and correlation coefficient.

(A) N2

(B) HI

(C) $N2^{m}$ and HI^{p}

(D) HI^{m} and $N2^{p}$

Figure S4

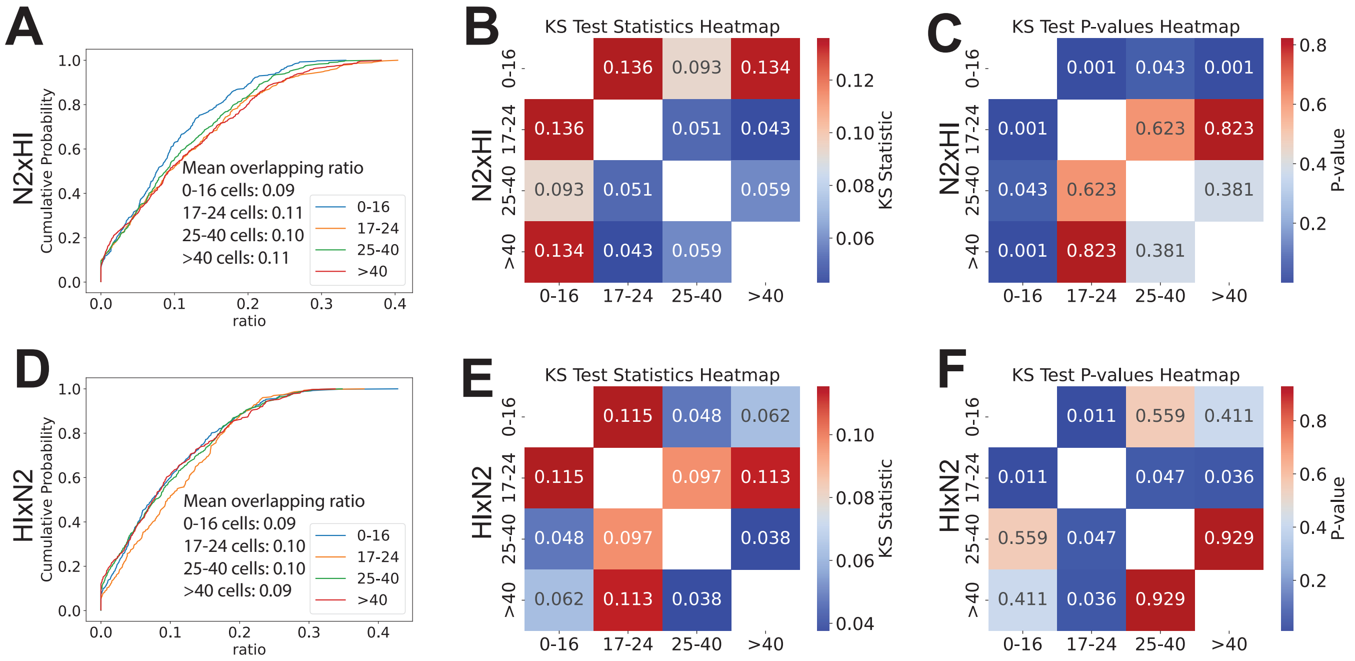

Figure S4

Kolmogorov-smirnov test for overlapping ratio in different development stages.

(A) Cumulative distribution curve of overlapping rate in different developmental stages in N2xHI embryos. Mean overlapping ratio of different stages indicated in the figure.

(B) Heatmap of Kolmogorov-smirnov statistic for every pair of developmental stages in N2xHI embryos.

(C) Heatmap of p-values of the Kolmogorov-smirnov test for every pair of developmental stages in N2xHI embryos.

(D) Cumulative distribution curve of overlapping rate in different developmental stages in HIxN2 embryos. Mean overlapping ratio of different stages indicated in the figure.

(E) Heatmap of Kolmogorov-smirnov statistic for every pair of developmental stages in HIxN2 embryos.

(F) Heatmap of p-values of the Kolmogorov-smirnov test for every pair of developmental stages in HIxN2 embryos.
