# Supplementary material for: Multiplex DNA fluorescence in situ hybridization to analyze maternal vs. paternal C. elegans chromosomes
Source: Genome Biol. 2024 Mar 14;25:71. doi: 10.1186/s13059-024-03199-6 (PMC10941459; doi:10.1186/s13059-024-03199-6)
Supplement: Supplementary file 4 — Additional file 4. Review history. [file 13059_2024_3199_MOESM4_ESM.docx]

Review History

**First round of review**

**Reviewer 1**

**Comments to author:**

Review Commons *Report 1*. <https://doi.org/10.15252/rc.2023238718>

**Reviewer 2**

**Comments to author:**

Review Commons *Report 2*. <https://doi.org/10.15252/rc.2023765496>

**Reviewer 3**

**Comments to author:**

Review Commons *Report 3*. <https://doi.org/10.15252/rc.2023548355>

**Authors Response**

**Point-by-point responses to the reviewers’ comments:**

Below is our itemized response to reviewers. We apologize for the delay in resending the new version – the first author’s father contracted cancer and has now died, and the author left the lab. These events slowed us up considerably. Nevertheless, we have made the requested changes, as outlined below.

We have added 5 new experiments/analyses requested by the reviewers:

-	We calculated the 95% confidence interval and used regression analysis for power law fitting in figures 2, 3, 4.

-	We performed statistical analysis (Chi-squared and Cramér’s V) in figures 2 and 5 for the strain crosses and co-cluster analysis.

-	We added a graph of cell stages to Supplemental figure 2.

-	We determined the nuclear sizes for the samples under study. See Supplemental figure 2.

-	We added statistical analysis (Pearson correlation) for the replicates (Supplemental figure 3).

Reviewer #1 (Evidence, reproducibility and clarity (Required)):

1) In figures 2-4, it is important to note what stages of embryos are being analyzed and whether any analysis was done to determine if the chromosomes varied with embryonic stage?

We have included new data showing the ages of the embryos understudy and described the embryos:

*All embryos - homozygous N2 or HI embryos or crosses were at or below the 40-cell stage (Supplemental Figure 2).*

2) The authors need to clearly define chromosomal step size, scaling coefficient and pair wise distance and then describe the difference between these measurements. For example, it would be nice in relation to figure 4F if it was described exactly what it means to have an increase in step size along with a decrease in scaling exponent. This will enable the reader to more easily interpret the results.

We now explain these parameters more clearly in Results.

*One method of quantitatively assessing a chromosome’s compaction and 3D path in nuclear space is to compare the genomic (2D) and spatial (3D) pairwise distance measurements using power-law fitting (Mirny 2011, Wang 2016, Sawh 2020). Power-law fitting takes into account the polymer nature of the chromosome (where regions close in genomic distance are expected to be close in spatial distance, and vice versa). We can derive metrics from the fit of the raw data to describe the polymer step size (compaction) and scaling exponent (3D path). The step size the average distance between the points along the chromosome. A smaller step size indicates a more compact chromosome. The scaling exponent of a random walk polymer is expected to be 0.5, while an ideal highly crumpled (fractal globule) polymer where all spatial distances are proportional to genomic distances will have a scaling exponent of 0.3. Scaling exponents ~0.2 indicate more intermixing between points with large genomic distances.*

3) Is there any way to determine if changes in the step size and scaling are significant? It would be good to know if the changes are actually significant.

We have calculated the 95% confidence limit and the statistical significance for figures 2, 3 and 4.

4) In figure 5 (and maybe in earlier figures as well), it would also be helpful to mark the different clusters in the figures to show what is meant by a folder arm or a compacted central domain and refer to every panel in the text (only some descriptions in the text reference specific panels).

We have added text to define these regions:

*The nascent B compartments (or “chromosome arms” at TADs 1-7 and 18-22) exhibit long-range folding, whereas the nascent A compartment, encompassing TADs 8-17, is more extended.*

5) In figure 6, the authors need to do a better job of describing how the data is being presented. The % overlap is being graphed by density, but density of what? Also, the text mentions the total number of nuclei that overlap, but how is that number derived from the presented data? Finally, the data are broken down by embryonic stage, but there is no mention of this in the text. It is not mentioned until the discussion. Overall, this makes it very difficult to determine what the data are showing.

This figure shows the frequency of overlap between HI and N2 by territory (A) and the distances between all points along the chromosome (B, C). We have rewritten the figure legend:

*Figure 6: (A) The proportion of chromosome pairs (“Density” y axis) is graphed with respect to the degree of territory overlap (% overlap), calculated as the fraction of voxels in the overlap region divided by the total voxels for both territories (x axis). Territory regions were calculated from the N2 and HI probes. N2 and HI chromosomes from crosses between N2^m^ and HIp in different age-groups of embryos indicated by color-coding.*

We have also moved the relevant paragraph about figure 6A from the Discussion to Results. Originally placed in the Discussion because of the conjecture, we agree that it is better suited in Results.

*Among all embryos scored for overlapping homologues, the transcriptionally inactive 2-4 cell stage embryos displayed the least. After the first zygotic genome activation (ZGA, 4-cell stage), embryos exhibited an increase in homologue overlap (Figure 6A, arrow). In other species, chromosomal regions of actively transcribed genes tend to localize together (Branco and Pombo 2006), suggesting that transcription might play a role in the increased interactions between homologs in C. elegans as well. Since we see an increase in overlap between homologs right after ZGA, when nuclear sizes are not very different (5-16cell), it is unlikely that decreased nuclear size is a driver of intermingling. An alternative hypothesis for increased chromosomal interaction in these very early stages could be differences in the local epigenetic landscape of very early embryos. Constitutive heterochromatin in the form of H3K9 methylation accumulates gradually in early C. elegans embryos (Mutlu, Chen et al. 2018). Thus, it is possible that the increased territory intermingling and heterochromatin formation are linked phenomena.*

6) In the discussion, the authors perhaps should spend more time interpreting their results in light of others work on the maternal and paternal inheritance of chromatin in C.

elegans. For example, Arico et al 2011 Plos Genetics from the Kelly Lab. In addition to examining chromatin in the early embryo, in this paper the authors examine translocations, which might be interesting to look at using the technique presented here. Also, a number of recent papers from the Strome lab have examined chromatin

inheritance from sperm. It would be interesting to interpret the finding that paternal chromosomes are influenced by the maternal environment, in light of this work.

We have added a new paragraph to discuss parental inheritance in C. elegans (references omitted here).

*What might account for sex specific differences? Chromatin bearing modified histones is inherited from C. elegans parents, including marks associated with germline transcription such as histone H3K4me2, histone H3K36me3 and histone H3.3, as well as marks associated with germline silencing, such as histone HTAS-1, H3K9me3 and H3K27me3. It is possible that differential modification of maternally vs. paternally donated chromosomes could account for differential compaction in descendants. For example, H3K9me3 and H3K27me3 are associated with chromatin compaction and are enriched on the X chromosome in the male germ line and early embryo. Conversely, it is possible that organization of the genome in sperm or oocytes influences gene expression in the progeny beyond local chromatin marks. This feature may extend beyond the X chromosome but it is currently unknown whether autosomes also show parent-of-origin differences. The N2/HI system described here can help resolve this issue in future studies, by providing a means to distinguish chromosomes from each parent.*

Reviewer #2 (Evidence, reproducibility and clarity (Required)):

1) Page 1: We suggest the authors broaden the reach of their introduction to include well-known examples outside of C. elegans of the impact of parent-of-origin on 3D genome organization. Such examples would include X-inactivation, selective silencing of paternal genomes, the physical elimination of paternal chromosomes, and the like.

We have added the following text (references omitted):

*Other species also show sex-specific, parental effects on chromatin regulation. Striking examples include differential expression of the IGF2-H19 locus in mammals, silencing of the paternal X chromosome in marsupials and the early mouse embryo, and loss of the paternal genome in the males of many arthropod species. DNA methylation, long non-coding RNAs and the Polycomb complex have been implicated in these processes, and give rise to altered chromosome looping and genome organization.*

2) Page 1: We also suggest the authors consider including mention of observations from the following research publications and review:

Mayer W et al. Spatial separation of parental genomes in preimplantation mouse embryos. 2003 PMC2169371

Reichmann J et al. Dual-spindle formation in zygotes keeps parental genomes apart in early mammalian embryos. 2018 PMID: 30002254

Nagele R, Freeman T, McMorrow L, Lee HY. Precise spatial positioning of chromosomes during prometaphase: evidence for chromosomal order. 1995 PMID: 8525379

Hua LL et al. Mitotic antipairing of homologous and sex chromosomes via spatial restriction of two haploid sets. 2018 PMID: 30530674

Hua LL, Casas CJ, Mikawa T. Mitotic antipairing of homologous chromosomes. 2022 PMC9731508

We have added many new references regarding homologue pairing and some of the interesting papers listed here, with a focus on interphase since those are the cells under study in this manuscript.

3) Page 5: The authors designed their strain-specific probes to target 172 insertions that are over 1 kb in size and distributed across ChrV. We ask the authors to describe these insertions in greater detail, especially as, later in the manuscript, the authors touch on the possibility that sequence differences between the two strains may account for differences in chromatin architecture. How many insertions were on the N2 and HI chromosomes, respectively? What is the range and distribution of insertion sizes for all insertions as well as specifically for the N2 and HI ChrV chromosome? Do the insertions contain repetitive sequences, or are they predominantly composed of unique sequences? What is their distribution with respect to genes, active regions, TADs? Is there an explanation for why some are clustered? If they contain genes, are the genes enriched in certain GO categories? Do the insertions differ in their characteristics across the different chromosomes? This information could be included as graphs and/or tables.

Please see Supplemental Figure 1 for information on insertions between the two strains, i.e. number, size and position. Many of the other interesting questions asked by the reviewer are beyond the scope of this paper as there are a lot of differences between the strains. We cite the pertinent papers.

4) Page 5: Given that N2 and HI ChrV chromosomes differ by the number of insertions and, ultimately, probes, how might these differences have skewed the authors' results, especially with respect to overlap between the homologs? Here, simulations of different ratios of insertions between N2 and HI could be clarifying.

The tracing probes fall in regions conserved between the two species ie we could visualize all the regions in both N2 and HI. It is the strain territories that are sensitive to the polymorphisms. That means a given strain territory might be larger or smaller, but that would not affect the trace itself. The strain territory is only for strain identification. We’ve tried to make this point more clearly:

A benefit of our method is that the probes are used to identify the chromosome territory associated with a particular strain, and not for tracing per se. Therefore, the tracing probes are not restricted to regions of the genome with strain specific differences, nor do they rely on sensitive differences in hybridization temperature due to single molecule polymorphisms.

5) What difficulties were encountered when tracing the paths of overlapping homologs, and how were these difficulties accounted for and/or solved? Did the different numbers and distributions of insertions between N2 and HI exacerbate the challenge? What confidence levels accompanied their findings?

For the most part, this was not a problem because the regions of overlap along the chromosomes were distinct. Therefore, we were able to trace using nearest neighbor assignments even with intertwined chromosomes.

We have included a section in the Methods to clarify how we categorized traces and the criteria used to exclude some traces from further analysis. In addition, we show what proportion of traces were excluded for what reason for traces coming from hybrid embryos.

*To account for wrongfully segmented and traced chromosomes in the high-throughput analysis, we added several drop-out criteria for traces (see below), which will be excluded from further analysis. This included the presence of more than 4 traces (or copies of the chromosome) in one nucleus and the presence of more than 2 traces per strain per nucleus, situations which are biological impossible in a wild-type setting. These typically arise due to over-segmentation of the territory. We assessed the efficiency of this method to classify traces accurately by counting how often traces were excluded due to these criteria and used for this assessment four out of six datasets of traces from N2mat:HIpat embryos. Out of the 1479 total traces 497 traces were classified as HI and 680 as N2. The remainder of 302 traces (or 20.4%) were excluded for following reasons: (1) For 34 traces (2.3%) any region was present more than once in the trace. (2) For 70 traces (4.7%) more than 4 traces were detected in one nucleus. This is caused by over segmentation of the primary probe signal, resulting in splitting of a traces. (3) For 32 traces (2.2%), there were equal to or less then 4 traces per nucleus, but more than 2 traces were classified as HI and (4) for 96 traces (6.5%), there were equal to or less then 4 traces per nucleus, but more than 2 traces were classified as N2. The ladder two are caused by inaccurate segmentation of the strain marking territories. (5) 70 (4.7%) Traces were equally close to the strain-marking territory for N2 and HI and therefore excluded.*

*Similarly for embryos of crosses between HI hermaphrodites and N2 males we excluded 654 (21.9%) out of 2982 of all traces. (1) 78 (2.6%) of traces contained the same regions twice.*

(2) *128 (4.3%) traces were excluded because 4 or more traces were detected in one nucleus.*
(3) *For 102 (3.4%) Traces more then 2 traces of HI were present in a nucleus and (4) for 179 (6%) traces more the 2 traces were assigned to N2 within the same nucleus. And (5) 167 (5.6%) traces were excluded because they could not be assigned to either N2 or HI.*
6) Page 4: It would be helpful if the authors to put their insertion-based method into the context of other studies that have developed and used FISH to distinguish homologs.

We clarify that others have used SNPs for DNA FISH and to identify the parent of origin e.g. POD FISH, but our approach is the first for chromosome tracing, and does not restrict the choice of tracing probes to regions with sequence difference. We now discuss this here (references omitted):

*Previous studies have distinguished chromosomes using DNA FISH, but to our knowledge this is the first adaptation of chromosome tracing to distinguish maternal vs. paternal chromosome conformations during development.*

*To analyze these hybrids accurately, we have designed and implemented strain-specific FISH probe sets, and developed an image analysis pipeline that accurately distinguishes the parental chromosomes in embryos. A benefit of our method is that the probes are used to identify the chromosome territory and not for tracing per se. Therefore, the tracing probes are not restricted to regions of the genome with strain specific differences, nor do they rely on sensitive differences in hybridization temperature due to single molecule polymorphisms.*

7) Pag 6: It would clarifying if the authors provided details about the mis-annotation of the Thompson genome and how it is pertains to probe design.

We state now within the main text: *This signal likely corresponds to a small region within the HI genome that was not included in the Thompson HI genome, which we used for our probe design, but was present in a later HI genome release, as revealed by a BLAST search (Thompson, Snoek et al. 2015, Kim, Kim et al. 2019) (see Methods)*

We included a section in the Methods section which clarifies this:

*For N2-specific probes after detecting a slight signal when hybridizing to HI embryos (****Figure 1E****), we used NCBI BLAST 2.9.0+ to blast the N2-specific probe library against the HI genome released by Kim et al., which was done using long read sequencing. We found 536 of the 5577 N2-specific probes for ChrV binding along the HI ChrV. 253 of which were located very close to each other within 9.5kb between 17.32 and 17.33Mb along ChrV of the Kim genome. We think that this concentration of probes results in the background staining in HI.*

8) Page 6: The authors state that "...N2 and HI...harbor sequence differences, some of which are predicted to affect chromatin architecture" and that HI lacks ppw-1. We ask the authors to provide a more thorough discussion. To what extent might such predictions rest on the insertional differences between the strains?

We have added the following text, but it’s hard to predict what the functional outcomes would be since they are not well described in either species, with the exception of *ppw-1:*

*For example, HI lacks the germline RNAi component ppw-1 [TIJSTERMAN 2002] and the sperm-expressed, selfish genetic element peel-1 [seidel 2008]. N2 and HI also differ in certain multi-gene families such as BATH factors (BTB/POZ + MATH domains) and nuclear hormone receptors [Thompson waterston]. The effects of all but ppw-1 are unknown. Since experimentally introduced siRNAs were shown to be sufficient to induce chromatin compaction, it is possible that the lack of germline RNAi in HI strains might influence chromosome conformation (Tijsterman, Okihara et al. 2002, Fields and Kennedy 2019). We therefore wanted to investigate if these two strains exhibited similar genome structures by comparing chromosomes from N2 or HI homozygous embryos.*

9) Page 7: How much smaller is the HI genome and what percent of this difference is due to insertions (deletions)? Related to this, how much smaller is the HI ChrV chromosome as compared to N2?

We have included now in the main text:

*These differences between N2 and HI may reflect the smaller size of the HI genome, which is about 2Mbs shorter in total then the N2 genome. This represents a difference of about 2%. In addition, the relative size difference between chromosomes from HI and N2 is in fact the largest for ChrV, with HI ChrV being 741kb shorter than the N2 ChrV, representing a difference of 3.5% (Thompson, Snoek et al. 2015).*

10) Page 7 and throughout: For those readers who have not read Sawh and Mango 2020 and Sawh et al. 2020, or who are unfamiliar with the broader category of imaging technologies that support the current study, we ask the authors to provide much more background and citations to key methods. Without this information, many readers will neither sufficiently understand the strategies used for imaging acquisition, processing, and analysis nor grasp the relevance of terms such as step size, polymer step size, power-law fitting, etc. and therefore be less able to assess the authors' data and conclusions.

We have added additional explanations to the Discussion and Results to clarify these points:

*Chromosome tracing uniquely allows direct visualization of many genomic regions in cells without disrupting the nuclear environment, and direct quantitative measurement of chromosome conformation (Steven review). The major limitation is that compared to sequencing-based biochemical approaches like Hi-C, tracing technology does not yet reach very high resolution while maintaining genome-wide capability. However, another strength is that chromosome tracing is also a single-molecule approach, which enables researchers to compare the copies of the same chromosome in each nucleus. Previous tracing studies have not been able to distinguish the parent-of-origin of each chromosome (Sawh, Wang, Jun Han, Alistair, Nollmann, Ting Wu papers), and this study provides this advance.*

*One method of quantitatively assessing a chromosome’s compaction and 3D path in nuclear space is to compare the genomic (2D) and spatial (3D) pairwise distance measurements using power-law fitting (Mirny 2011, Wang 2016, Sawh 2020). Power-law fitting takes into account the polymer nature of the chromosome (where regions close in genomic distance are expected to be close in spatial distance, and vice versa). We can derive metrics from the fit of the raw data to describe the polymer step size (compaction) and scaling exponent (3D path). A smaller step size indicates a more compact chromosome. The scaling exponent of a random walk polymer is expected to be 0.5, while an ideal highly crumpled (fractal globule) polymer where all spatial distances are proportional to genomic distances will have a scaling exponent of 0.3. Scaling exponents ~0.2 indicate more intermixing between points with large genomic distances.*

11) General statement: When the authors infer similarity or differences between power-law fittings, scaling exponents, step sizes, etc. what is the statistical significance of those comparisons?

We have added statistics for figures 2-4 to clarify this issue.

12) Experiments in general: We suggest the authors provide considerably more quantitation. For example, we urge them to provide the number of trials, sample sizes, numbers of embryos examined for all experiments. Equally important would be information regarding the stage of embryos examined and, where more than one embryonic stage was involved, the number of embryos for each stage. Are there stage-specific changes? We are also concerned about the impact of mixed populations of embryos on studies using unsupervised clustering. In other words, what was the contribution of developmental stage to the outcome of the clustering? Furthermore, if not all nuclei in an embryo were captured, we ask the authors to give the percent of nuclei captured from an embryo and reasons why only a subset of nuclei were

included	in	the	analysis.

- We have added more statistics for Figures 2-4.
- We have clarified details on trials, number of embryos etc. in Methods.
- We have added information on the embryonic stages to Supplemental Figure 2 (all embryos were 40 cells or younger).
- The question of developmental stage and its effects on clustering are unlikely to influence the conclusions here, based on prior studies from Sawh and Mango that saw effects only late. These embryos are early (<40 cell stage) when we envisioned parental effects would be strongest.
- We performed statistical analysis of replicas
- We have added 95% confidence intervals for the power law polymer fitting.

13) With regard to cluster analyses both here and elsewhere, will the authors please include statements of statistical significance whenever they note differences and/or similarities?

Please see above.

14) Page 8: It would be helpful if the authors explained how they implemented the nearest-neighbor approach, including caveats and limitations (success rates, drop-out rates, etc.) and providing statistical assessment wherever possible.

We have added an explanation to Results:

*The underlying assumption using this approach is that we assume that region ‘n’ along the chromosome connects immediately to the closest focus in 3D space that was detected for region ‘n+1’, and not another detected ‘n+1’ focus (which we assume belongs to another chromosome). This approach agrees well with simulated polymer models (Jia et al., 2023), however there is no absolute way to assess what the ‘real’ chromosome path is, and therefore it is not possible to calculate our success rate.*

15) Page 8: "In some instances (6-8% of traces), traces were ambiguous and excluded from further analysis". We ask the authors to provide more detail. For example, what does ambiguous mean and, with respect to the 6-8% value, what was the total number of traces? What was the distribution of all traces (prior to filtering) with respect to percent of targets detected? Also, how coincident were the two homologs of a nucleus to each other in terms of capturing all the targets?

We used parameters to what has been used before (Sawh et al. 2020) to remove traces where, for example, the chromosome forked due to close proximity of nearest neighbors, or due to DNA replication. High background was another reason. And if there was a ‘tie’ between N2 and HI designation, that traces was also not considered. All of these examples were quite rare.

We included a section in the manuscript to clarify how we categorize traces and based on which criteria they were excluded from further analysis. In addition we show what proportion of traces were excluded for what reason for traces coming from hybrid embryos.

*To account for wrongfully segmented and traced chromosomes in the high-throughput analysis, we added several drop-out criteria for traces (see below), which will be excluded from further analysis. This included the presence of more than 4 traces (or copies of the chromosome) in one nucleus and the presence of more than 2 traces per strain per nucleus, situations which are biological impossible in a wild-type setting. We assessed the efficiency of this method to classify traces accurately by counting how often traces were excluded due to these criteria and used for this assessment four out of six datasets of traces from N2mat:HIpat embryos. Out of the 1479 total traces 497 traces were classified as HI and 680 as N2. The remainder of 302 traces (or 20.4%) were excluded for following reasons: (1) For 34 traces (2.3%) any region was present more than once in the trace. (2) For 70 traces (4.7%) more than 4 traces were detected in one nucleus. This is caused by over segmentation of the primary probe signal, resulting in splitting of a traces. (3) For 32 traces (2.2%), there were equal to or less then 4 traces per nucleus, but more than 2 traces were classified as HI and (4) for 96 traces (6.5%), there were equal to or less then 4 traces per nucleus, but more than 2 traces were classified as N2. The ladder two are caused by inaccurate segmentation of the strain marking territories. (5) 70 (4.7%) Traces were equally close to the strain-marking territory for N2 and HI and therefore excluded.*

*Similarly for embryos of crosses between HI hermaphrodites and N2 males we excluded 654 (21.9%) out of 2982 of all traces. (1) 78 (2.6%) of traces contained the same regions twice.*

(2) *128 (4.3%) traces were excluded because 4 or more traces were detected in one nucleus.*
(3) *For 102 (3.4%) Traces more then 2 traces of HI were present in a nucleus and (4) for 179 (6%) traces more the 2 traces were assigned to N2 within the same nucleus. And (5) 167 (5.6%) traces were excluded because they could not be assigned to either N2 or HI.*
16) Page 8: "...we classified traces into N2 or HI based on whether the trace was located closest to a strain marking volume for N2 or HI." Will the authors please quantify "closest" and explain what this means, whether there was a cut-off and, if there had been, how it was determined and implemented? What percent of cells were problematic, and were there traces that did not overlap the strain marking volumes at all? As stated in the Materials and Methods, only a subset of traced chromosomes were analyzed for overlap - why were only a subset analyzed for overlap, how was the subset selected, and how many/what percentage did these traces represent? It would also be helpful if the authors provided a quantitative summary of the traces.

For each region within a trace we calculated the smallest Euclidian distance between the boundary points of the strain marking volumes for N2 and HI. If the region was based on this calculation closer to N2 it was classified as N2 and vice versa for HI.

The signal of the tracing probes overlapped the strain territory probe, but not all spots along the trace were enclosed in the strain probes. We used a majority call for the territory. There was no cut off.

17) Page 8: Did the authors account for chromatic aberration and, if so, what protocol did they use?

Yes we accounted for chromatic aberration and we explain that in the Methods section now:

*Chromatic aberration was corrected by creating a transformation function in x y and z of the 647nm signal into the 560nm signal (Wang 2016, Sawh 2020). This function was calculated using images of multicolor 100nm Tetraspeck beads and aligning beads in the 647 channel to the same beads in the 560 channel. In addition, were visualized after each round of imaging to ensure microscope alignment between rounds of hybridization.*

18) Page 8: "...counting how often more than two N2 or HI traces were detected in one nucleus." This is puzzling, and we suggest that the authors include explanations for how this might have happened. Did the nuclei not contain signal from the other strain marking probe at all? Was there a bias for this to happen with N2 or HI chromosomes? Could this have been a consequence of biology or of the algorithm for tracing?

This likely reflects over segmentation of territories, leading to a chromosome being “split up”. We included a section in the Methods that clarifies this problem and its frequency.

The authors' observations are reminiscent of the many implications raised by Jia et al. (2023; A spatial genome aligner for resolving chromatin architectures from multiplexed DNA FISH. PMID: 36593410), and we ask the authors to comment on the relevance of their observations to those in this recent publication.

Our paper does not discuss sister chromatids after replication – we apologize for the confusion. We cite the paper above in that regard and also for the polymer simulation discussion:

*The underlying assumption using this approach is that region ‘n’ along the chromosome connects to the closest focus in 3D space that was detected for region ‘n+1’, and not a more distant ‘n+1’ focus, which we assume belongs to another chromosome. This approach agrees well with simulated polymer models (Jia et al., 2023), however there is no way to tell what the ‘real’ chromosome path is, and therefore it is not possible to calculate our success rates.*

*In Drosophila and other Dipterans, homologous chromosomes are paired in somatic cells (McKee 2004) and 20% of alleles show pairing for genes undergoing transvection (e.g. gypsy [[6]]). Up until now, strict pairing of homologs in other organisms has not been observed. In mammals, sister chromatids pair after replication [jia ren 2023], and homolog pairing is tissue specific and restricted to particular regions of chromosomes (Apte and Meller 2012).*

19) Page 8: "We found only a minority of 2% of HI traces and 7% of N2 traces were mis-assigned and excluded these from downstream analysis." 2% and 7% of what total?

We changed this section and included more detailed quantitation of how many traces were excluded and for what reason. Please see Methods.

20) Page 8: How do pairwise distances remain almost identical between N2 and HI and yet generate different scaling exponents?

The scaling exponent differences are relatively small for the N2^m^ and HIp cross. We see more fluctuation for the converse, which we discuss in the Results and Abstract.

21) Page 9: Figure 1F shows images of embryos derived from N2 hermaphrodites x HI males. It would be helpful if the authors added analogous images from the reciprocal cross as a supplementary figure.

Done. Please see Supplemental Figure S1

22) Page 2, 9, 9, and 12: The authors make several comments regarding the action of factors

in trans:

"... factors from the mother impact chromosome folding in trans (p. 2)"; "... the Hip decompacts when subjected to the N2m environment and implies that the paternal chromosome is influenced by the maternal environment in trans (p. 9)"; "...N2 chromosomes influence HI chromosomes in trans, while N2 chromosome structure seems to be resistant to influences by the HI chromosomes (p. 9)"; and "...implicating maternal factors that act in trans (p. 12).

While provocative, these statements call for more concrete consideration. Are the authors using "in trans" in lieu of "indirectly", or are they alluding to factors, such as ppw-1, or direct physical contact? Without further substantiation or argument, mention of in trans activity might best be reserved for the Discussion.

We have rewritten these sections to remove the word ‘factors’ and clarify that it is the effect of the maternal environment. For example

*The data indicate that the maternal environment impacts chromosome folding in trans.*

We don’t know what in the maternal environment is responsible for the effects we see, but since the mother affects the HI chromosome, by definition it is acting in *trans*. We do not speculate on what in the maternal environment affects the chromosome because we have no evidence to support one notion over another.

23) Page 10: "While HIp subpopulations were characterized by folding of one or the other chromosome arm, N2p clusters were more open and a subpopulation with a highly folded right arm was not present" (Figure 5CD). Was there a significant correlation between left vs. right arm folding and overall genome organization and function?

In our previous work we showed that the left or the right arm could be more/less compacted independent of the other. Please see Sawh et al., Mol Cell, 2020. The biggest effect on folding was attachment to/detachment from the lamina.

24) Page 11: Will the authors please provide a more explicit definition of alignment as well as a more detailed description of how alignment is quantified in the main text?

We are not sure which alignment is being referred to. For tracing we now explain:

*Chromatic aberration was corrected by creating a transformation function in x y and z of the 647nm signal into the 560nm signal (Wang 2016, Sawh 2020). This function was calculated using images of multicolor 100nm Tetraspeck beads and aligning beads in the 647channel to the same beads in the 560 channel. In addition, the beads were visualized after each round of imaging to ensure microscope alignment between rounds of hybridization.*

For chromosomes, we now state:

*Sequences that promote transvection stabilize the association between alleles, leading to a significant proportion of aligned homologues within 300-400nm of one another (e.g. 20% for gypsy)* Click or tap here to enter text.*.*

*Distances were generally fairly large, on the order of 2.5 µm in nuclei that are on average 5.3 µm in diameter. While this absolute distance may be skewed by fixation conditions, this result suggests that although homologous chromosomes frequently overlap a portion of their territories, homologous genes are not more closely aligned with one another than with other points along the chromosome. We also note that there were few significant differences for chromosomes from N2 vs HI.*

25) Page 11: With respect to direct physical contact, the authors mention transvection, which they conclude in the abstract is unlikely because pairing between homologs was observed to be rare. As transvection and pairing can both be short-lived, and the data are not compelling, the statement may need to be toned down considerably and/or be moved to the discussion.

We have clarified the transvection effect as follows:

*In Drosophila and other Dipterans, homologous chromosomes are paired in somatic cells (McKee 2004) and 20% of alleles show pairing for genes undergoing transvection (e.g. gypsy [[6]]). Up until now, strict pairing of homologs in other organisms has not been observed. In mammals, sister chromatids pair after replication (Jia 2023), and homolog pairing is tissue specific and restricted to particular regions of chromosomes (Apte and Meller 2012).*

*In this study, we found that in C. elegans homologs of ChrV frequently intermingle, but homologous regions rarely closely align. Given the large number of chromosomes sampled (in the thousands) 10% or 20% paired chromosomes would have been easily detected.*

26) Page 11: The authors draw a distinction between % territory overlap and physical distances between homologs. In particular, the differences between % overlap across different stages is quite interesting and potentially suggests embryo stage-specific changes. Could the authors explore this further by breaking down mean pairwise distances into different embryo stages (Figure 6B and C)?

The only breakdown is shown in Figure 6A

27) Pages 2, 11-13: When the authors use "sisters", do they mean "homologs"? If the latter, we recommend they use "homologs", only, as "sisters" refers to the sister chromatids after replication. If, however, the authors are using "sisters" to mean sister chromatids, will they please explain how their data can distinguish sisters?

We apologize! We have changed the wording to homologues throughout.

Discussion: We encourage the authors to speculate further regarding the basis of decompaction. Is it a hybrid-specific phenomenon?

We have added a new paragraph to the Discussion to discuss the difference we see between the strains (references omitted):

*What might account for sex specific differences? Chromatin bearing modified histones is inherited from C. elegans parents, including marks associated with germline transcription such as histone H3K4me2, histone H3K36me3 and histone H3.3, as well as marks associated with germline silencing, such as histone HTAS-1, H3K9me3 and H3K27me3. It is possible that differential modification of maternally vs. paternally donated chromosomes could account for differential compaction in descendants. For example, H3K9me3 and H3K27me3 are associated with chromatin compaction and are enriched on the X chromosome in the male germ line and early embryo. Conversely, it is possible that organization of the genome in sperm or oocytes influences gene expression in the progeny beyond local chromatin marks. This feature may extend beyond the X chromosome but it is currently unknown whether autosomes also show parent-of-origin differences. The N2/HI system described here can help resolve this issue in future studies, by providing a means to distinguish chromosomes from each parent.*

Minor comments:

29) Page 1: Although the introduction focuses on C. elegans, the genome length that is mentioned (2 meters) is more aligned with that of mammalian species. The authors could cite a range of lengths or make more clear which species is being discussed.

Thanks for the comment. We have rewritten this section as follows:

*C. elegans nuclei face a complex organizational problem, whereby 100 Mb of genomic DNA is packaged into a confined space as small as 1-µm in diameter*

30) Page 5: As the figures label the strains as Hawaiian and Bristol, the authors might wish to include this nomenclature in the main text. Curiously, the authors use several different spellings for the Hawaiian/Hawai'ian/Hawaiin/Hawaii strain.

Yes, we’ve corrected this error. Thanks for pointing it out.

31) Page 5: Will the authors please explain why the common whole-chromosome tracing probes have only one tail, while the strain-specific probes have two tails, as shown in Figure 1D?

We started tracing with single tails. They work, but a double tail generates a brighter signal and, in some cases, helps visualize regions where there is lower probe coverage. For example:

“... *since for some regions along Chr V strain-marking probes were scarce due to a lack of strain-specific insertions, we decided to equip each N2-specific and each HI-specific probe with two binding sites for fluorescently labeled secondary oligos to increase the signal***.**

32) Figures in general: The axes of a number graphs and heat maps need to be labeled. Done
33) Materials and Methods: The section on "Cluster analysis" is missing units for the resolution.

There are no units for resolution, which is used to define the granularity of the clusters. This is a trial-and-error approach to determine the depth of clustering in Seurat. Seurat recommends, for RNA, a value between 0.4-1.2. Resolutions chosen were as per Sawh et al, 2020 and focused on Clusters appearing different from one another. Over clustering tends to generate a lot of clusters that look the same.

34) Page 8: Will the authors please give a reference for and explain watershed segmentation?

We have added this clarification:

*Watershed segmentation is a method of image processing that automatically separates individual elements in the foreground (i.e. nuclei or chromosome territories) from the background (i.e. cytoplasm or nucleoplasm). We implemented watershed segmentation in*

*MATLAB	as	done	previously	(Sawh	Mol	Cell	2020)*
[*(https://www.mathworks.com/help/images/marker-controlled-watershed-segmentation.html)*](https://www.mathworks.com/help/images/marker-controlled-watershed-segmentation.html)*.*

i) present a more thorough summary of what is known about homolog positioning across species

We have incorporated more discussion on this topic (references omitted here):

*Other species also show sex-specific, parental effects on chromatin regulation. Striking examples include differential expression of the IGF2-H19 locus in mammals, silencing of the paternal X chromosome in marsupials and the early mouse embryo, and loss of the paternal genome in the males of many arthropod species. DNA methylation, long non-coding RNAs and the Polycomb complex have been implicated in these processes, and give rise to altered chromosome looping and genome organization.*

*In Drosophila and other Dipterans, homologous chromosomes are paired in somatic cells (McKee 2004) and 20% of alleles show pairing for genes undergoing transvection (e.g. gypsy [[6]]). Up until now, strict pairing of homologs in other organisms has not been observed. In mammals, sister chromatids pair after replication, and homolog pairing is tissue specific and restricted to particular regions of chromosomes. Earlier studies in mice using FISH demonstrated that chromosomes tend to locate in heterologous neighborhoods and work in human cells interestingly found larger distances between homologs then heterologues. These observations imply that inter-homolog interactions may be rare beyond dipterans. In this study, we found that in C. elegans homologs of ChrV frequently intermingle, but homologous regions rarely closely align. Given the large number of chromosomes sampled (in the thousands) 10% or 20% paired chromosomes would have been easily detected.*

, ii) describe how homologs have been previously distinguished by FISH and, thus, more clearly elucidate the specific advances they have enabled,

We have added more text to this effect:

*Previous studies have distinguished chromosomes using DNA FISH, but to our knowledge this is the first adaptation of chromosome tracing to distinguish maternal vs. paternal chromosome conformations during development.*

*Analysis of parent-of-origin effects typically relies on sequence differences between alleles. RNAs or genes bearing polymorphisms are used to distinguish the parental origins of the two alleles. One downside of this approach is that loci that lack polymorphisms cannot be studied, which restricts analysis to a smaller cohort of genes. Another downside is that sequencing errors can generate inaccurate estimates of biased allele expression. Here we describe an approach that circumvents these difficulties and enables parental identification to study chromosome organization.*

*A benefit of our method is that the probes are used to identify the chromosome territory and not for tracing per se. Therefore, the tracing probes are not restricted to regions of the genome with strain specific differences, nor do they rely on sensitive differences in hybridization temperature due to single molecule polymorphisms.*

iii) ground their work in more rigorous quantitation,

We have added additional statistics and 95% confidence information.

iii) provide a better description of the technologies (strengths as well as limitations) carried over from their previous studies so that readers can better evaluate the current study.

We have added this text:

*Chromosome tracing uniquely allows direct visualization of many genomic regions in cells without disrupting the nuclear environment, and direct quantitative measurement of chromosome conformation. The major limitation is that compared to sequencing-based biochemical approaches like Hi-C, tracing technology does not yet reach very high resolution while maintaining genome-wide capability. However, another strength is that chromosome tracing is also a single-molecule approach, which enables researchers to compare the copies of the same chromosome in each nucleus. Previous tracing studies have not been able to distinguish the parent-of-origin of each chromosome,, and this study provides this advance.*

**Reviewer #3** (Evidence, reproducibility and clarity (Required)):

statistical measures of significance. This is particularly important for the clustering and scaling analysis. This makes interpretation of the claims made in the text impossible.

We have added addition statistics for figures 2-4.

Throughout the manuscript (including in the abstract), the authors use the term "sister chromosomes" to (presumably) refer to the maternal and paternal chromosomes. This is a confusing term, since "sister chromatids" usually refers to the identical products of DNA replication, and "homologous chromosomes" is usually used to describe the parental chromosomes. The term would ideally be changed, and at the very least, it should be clearly defined.

Apologies. We have corrected the term throughout.

The authors could include a blurb on the methodology behind each data type to help the manuscript reach a larger audience.

This needs to be throughout the manuscript text, ‘each data type’ is everything, so I cannot address this in a stand-alone paragraph. When the revised text is ready I can make additions if necessary.

We have added considerably more information about the methodology and what it means. Here are examples:

*One method to assess a chromosome’s compaction and 3D path in nuclear space is to compare the genomic (2D) and spatial (3D) pairwise distance measurements using power-law fitting. Power-law fitting takes into account the polymer nature of the chromosome (where regions close in genomic distance are expected to be close in spatial distance, and vice versa). We can derive metrics from the fit of the raw data to describe the polymer step size (compaction) and scaling exponent (3D path). The step size is the average distance between the points along the chromosome. A smaller step size indicates a more compact chromosome. The scaling exponent of a random-walk polymer is expected to be 0.5, while an ideal highly crumpled (fractal globule) polymer where all spatial distances are proportional to genomic distances will have a scaling exponent of 0.3. Our prior studies found that C. elegans chromosomes had a scaling exponent of ~0.2, which reflects more intermixing between points with large genomic distances.*

*Watershed segmentation is a method of image processing that automatically separates individual elements in the foreground (i.e. nuclei or chromosome territories) from the background (i.e. cytoplasm or nucleoplasm). We implemented watershed segmentation using*

*MATLAB	as	performed	previously	(Sawh	Mol	Cell	2020)*
[*(https://www.mathworks.com/help/images/marker-controlled-watershed-segmentation.html)*](https://www.mathworks.com/help/images/marker-controlled-watershed-segmentation.html)*.*

*This step defined the volumes in which chromosomes were traced using a nearest-neighbor approach (****Figure 3A step 2; (Sawh, Shafer et al. 2020)****). The underlying assumption using this nearest neighbor aproach is that region ‘n’ along the chromosome connects to the closest focus in 3D space that was detected for region ‘n+1’, and not a more distant ‘n+1’ focus, which we assume belongs to another chromosome. This approach agrees well with simulated polymer models [jia bing ren 2023 Nature Biotechnology], however there is no way to tell what the ‘real’ chromosome path is, and therefore it is not possible to calculate success rates or drop-out rates.*

*Between 6-8% of traces were ambiguous and excluded from further analysis, similar to Sawh et al. These included traces where the chromosome forked due to close proximity of nearest neighbors, DNA replication/ multiple signals, high background, or if there was a ‘tie’ between an N2 vs. HI designation. All of these examples were quite rare.*

*The chromosome tracing method uniquely allows direct visualization of many genomic regions in cells without disrupting the nuclear environment, and direct quantitative measurement of chromosome conformation without the need for DNA ligation (Steven wang review). The major limitation is that compared to sequencing-based biochemical approaches like Hi-C, tracing technology does not yet reach very high resolution while maintaining genome-wide capability. However, another strength is that chromosome tracing is also a single-molecule approach, which enables researchers to compare the copies of the same chromosome in each nucleus. Previous tracing studies have not been able to distinguish the parent-of-origin of each chromosome, and this study provides this advance.*

The pipeline, meaning, and potential caveats of the clustering analysis should also be explicated.

The cluster analysis is exactly as published in Sawh et al., Mol Cell, 2020. One parameter about cluster analysis is to check the cluster against single molecules. We find that some single molecules resemble the cluster, but others do not look exactly the same. Nevertheless, the cluster analysis enables us to identify the prevalent folding patterns.

We have written:

*Within each chromosome trace, the distances between all possible pairs of regions were measured in three dimensions. For each region, we then averaged the observed distance for all traced chromosomes, and this average distance is displayed in the heatmap matrices.*

The implications of the map could also be better explained (e.g., that the red cluster means relatively looser regions, and the blue means more tightly compacted ones).

This information is listed in the figure legends.

The last sentence in the Introduction does not make clear sense. How does the similarity between N2 & HI open up the possibility of interrogating inheritance effects?

We have cut the phrase from the introduction.

1) Measure the nucleus diameter in N2/HI hybrid and HI/N2 hybrid.
   This information has been added to Supplemental Figure 2
2) Normalize the spatial distance to the nucleus size, rather than directly using the distance.

We do not have an algorithm that can adjust the distance between two probes dependent on the volume of the nucleus. This would be complicated to code, particularly since the nuclei are not spheres. We have opted for direct measurements for now.We also note that nuclear size may reflect chromosome compaction and therefore if we normalize, we may lose some of the phenomena that we are trying to study.

3) Explore some of the patterns in the spatial distance plots (e.g., red/blue lines and boxes). Are the sequences that are in them any different between N2 and HI in a way that might be able to account for these patterns?

This is unfortunately beyond the scope of this manuscript but would be a fascinating avenue to pursue in the future.

**Second round of review**

**Reviewer 1**

This is an excellent example of how reviewer commons should work. The authors did an outstanding job of responding to the original comments. The manuscript is now significantly improved because of it.

Overall, the authors have applied a difficult novel technique (chromosome tracing) to an interesting problem (distinguishing maternal vs paternal chromosomes) and made some important observations (that there is a maternal influence on the paternal chromosome and homologs don't align)

**Reviewer 3**

The revised manuscript by Gutnik, Mango and colleagues is improved with respect to the original manuscript (reviewed through Review Commons). Specifically, many of the claims have been backed by statistical analysis, and helpful explanations and diagrams have improved the accessibility of the manuscript. There are still a few cases where the text overstates the data or is imprecise. These are noted below in order of appearance. (Line numbers were not properly aligned with the set in the version provided.) As could be appreciated from the specific comments, the major remaining issue is the analysis of the data in Figure 6A and the related text.

Page 3, lines 31-32: Isn't this work also relies on sequence differences between alleles? The sentence makes it sound that this work somehow uses a different approach.

Page 3, lines 45-46: This line makes it sound like genome packaging is a nematode-specific problem, while it is universal.

Page 5, lines 10-12: The second point is weirdly worded; do the author mean that there are well-assembled sequenced genome for these strains?

Page 6, line 31: I think the authors meant to use the term "proof of concept".

Page 6, lines 52-54 and Figure 1E: The slight signal is not apparent in the figure. I wonder if an over-exposed image (perhaps in a supplementary figure) would be helpful to convey this point.

Page 8, lines 25-29: The difference in step size, as far as I can tell, were not statistically significant. Such non-statistically-significant differences should be noted clearly in the text.

Page 12, line 56: Should be "transcriptional".

Page 13: The discussion related to Figure 6A lacks in statistical analysis. This point is crucial and affects almost all interpretation. This relates both to comparisons within the data (e.g., lines 33-35, "After ZGA, embryos exhibited an increase in homologue overlap") - all such claims should be backup up by statistical analysis or removed from the text. The bigger issue is the lack of the basal level of randomized interactions. In other words, considering the size of the territories and the size of the nucleus, what is the chance that two unrelated chromosomes touch each other? Without an experimental assessment of such 'random' interactions, statements about the "43% [of nuclei] had an overlap" are impossible to be appreciated and are quite meaningless.
